# Supplementary material for: Sex differences in the association between visceral adiposity index and biological aging: A cross-sectional analysis of NHANES 1999–2018 with mediation by insulin resistance
Source: PLoS One. 2025 Sep 29;20(9):e0333472. doi: 10.1371/journal.pone.0333472 (PMC12478895; doi:10.1371/journal.pone.0333472)
Supplement: S1 Table — (DOCX) [file pone.0333472.s001.docx]

**Supplementary Information**

**S1 Table. Formulae for KDMAge.**

| **Component** | **Description** |
| --- | --- |
| Formula |  |
| Key Parameters | KDMAge is derived from a series of regressions of individual bio markers on chronological age in a reference population. The equation takes information from an n number of regression lines of chronological age (CA) regressed on n biomarkers. X is the value of the biomarker i measured for an individual. For each biomarker i, k, q, and s represent the regression intercept, slope, and root mean squared error, respectively. sBA is a scaling factor equal to the square root of the variance in chronological age explained by the biomarker set in the reference sample. |
| Reference Population | NHANES III nonpregnant participants aged 30–75 years. Algorithm parameters were estimated separately for males and women. |
| Biomarkers | Ten biomarkers based on previous studies, namely lymphocyte percentage, systolic blood pressure, total cholesterol, serum albumin, blood urea nitrogen, creatinine, mean corpuscular volume, glycated hemoglobin, white blood cell count, and alkaline phosphatase. |
| Implementation | BioAge package (<https://github.com/dayoonkwon/BioAge>) |
