# Supplementary material for: Sex differences in the association between visceral adiposity index and biological aging: A cross-sectional analysis of NHANES 1999–2018 with mediation by insulin resistance
Source: PLoS One. 2025 Sep 29;20(9):e0333472. doi: 10.1371/journal.pone.0333472 (PMC12478895; doi:10.1371/journal.pone.0333472)
Supplement: S2 Table — (DOCX) [file pone.0333472.s002.docx]

**Supplementary Information**

**S2 Table. Definitions of clinical covariates.**

| **Covariates** | **Abbreviation** | **Diagnostic Criteria** | **Data Source** | **References** |
| --- | --- | --- | --- | --- |
| Hypertension | HTN | The diagnosis is based on one of: | Physical examination + Medical history questionnaire | PMID: 39834472 |
|  |  | 1) Systolic blood pressure > 140 mmHg or diastolic blood pressure > 90 mmHg |  |  |
|  |  | 2) Physician-confirmed diagnosis |  |  |
| Cardiovascular Disease | CVD | The diagnosis is based on one of: | Medical history questionnaire | PMID: 40022176 |
|  |  | 1) Congestive heart failure |  |  |
|  |  | 2) Coronary artery disease |  |  |
|  |  | 3) Myocardial infarction |  |  |
|  |  | 4) Angina pectoris |  |  |
|  |  | 5) Stroke |  |  |
| Diabetes Mellitus | DM | The diagnosis is based on one of: | Laboratory tests + Medical history questionnaire | PMID: 39696515 |
|  |  | 1) Glycated hemoglobin ≥ 6.5% |  |  |
|  |  | 2) Fasting glucose ≥ 126 mg/dL |  |  |
|  |  | 3) 2-h oral glucose tolerance test ≥ 200 mg/dL |  |  |
|  |  | 4) Current use of antidiabetic agents |  |  |
|  |  | 5) A previous diagnosis by a physician |  |  |
| Chronic Kidney Disease | CKD | The diagnosis is based on one of: | Laboratory tests | PMID: 40177175 |
|  |  | 1) Estimated glomerular filtration rate < 60 mL/min/1.73 m² (CKD-EPI equation) |  |  |
|  |  | 2) Urine albumin-creatinine ratio > 30 mg/g |  |  |
| Cancer | - | Cancer is defined as having been told by a doctor or other healthcare professional that a malignant neoplasm has been diagnosed. | Medical history questionnaire | PMID: 40022176 |
