# Supplementary material for: Sex differences in the association between visceral adiposity index and biological aging: A cross-sectional analysis of NHANES 1999–2018 with mediation by insulin resistance
Source: PLoS One. 2025 Sep 29;20(9):e0333472. doi: 10.1371/journal.pone.0333472 (PMC12478895; doi:10.1371/journal.pone.0333472)
Supplement: S3 Table — (DOCX) [file pone.0333472.s003.docx]

**Supplementary Information**

**S3 Table. Mediating effects of diabetes mellitus between VAI and biological aging among the whole population.**

|  | **β (95% CI)** | ***P*-value** | **Mediation Proportion (%)** |
| --- | --- | --- | --- |
| VAI–KDMAge |  | | |
| Indirect | 0.0664 (0.0092–0.0965) | 0.022 | 8.69 |
| Direct | 0.6975 (0.5558–0.7967) | <0.001 |  |
| Total | 0.7639 (0.5864–0.8573) | <0.001 |  |
| VAI–KDMAgeAccel risk |  | | |
| Indirect | 0.0014 (0.0001–0.0020) | 0.022 | 5.51 |
| Direct | 0.0245 (0.0171–0.0258) | <0.001 |  |
| Total | 0.0259 (0.0180–0.0269) | <0.001 |  |

The models were adjusted for age, sex, race, education, marital status, poverty status, smoking status, alcohol consumption, M/VPA, HTN, CVD, cancer, and CKD.

VAI, visceral adiposity index; KDMAge, Klemera-Doubal method age; KDMAgeAccel, KDMAge acceleration; CI, confidence interval.
