# Supplementary material for: Sex differences in the association between visceral adiposity index and biological aging: A cross-sectional analysis of NHANES 1999–2018 with mediation by insulin resistance
Source: PLoS One. 2025 Sep 29;20(9):e0333472. doi: 10.1371/journal.pone.0333472 (PMC12478895; doi:10.1371/journal.pone.0333472)
Supplement: S4 Table — (DOCX) [file pone.0333472.s004.docx]

**Supplementary Information**

**S4 Table. Sex-specific associations of VAI quartiles with biological aging.**

| **VAI** | **Q1** | **Q2** | **Q3** | **Q4** | ***P*-value** |
| --- | --- | --- | --- | --- | --- |
| Age (years) |  | | | | |
| Female CA | 41.0 | 45.0 | 48.0 | 52.0 | <0.001 |
| Female KDMAge | 33.1 | 37.9 | 42.2 | 49.1 | <0.001 |
| Female KDMAgeAccel | -7.1 | -6.4 | -4.9 | -1.1 | <0.001 |
| Male CA | 41.0 | 45.0 | 46.0 | 46.0 | <0.001 |
| Male KDMAge | 32.0 | 36.4 | 38.7 | 42.3 | <0.001 |
| Male KDMAgeAccel | -9.4 | -8.3 | -6.7 | -3.1 | <0.001 |
| KDMAgeAccel risk (%) |  | | | | |
| Female | 25.9 | 29.0 | 36.6 | 46.4 | <0.001 |
| Male | 25.0 | 28.5 | 33.6 | 41.7 | <0.001 |

VAI, visceral adiposity index; CA, Chronological age; KDMAge, Klemera-Doubal method age; KDMAgeAccel, KDMAge acceleration.
