# Supplementary material for: Sex differences in the association between visceral adiposity index and biological aging: A cross-sectional analysis of NHANES 1999–2018 with mediation by insulin resistance
Source: PLoS One. 2025 Sep 29;20(9):e0333472. doi: 10.1371/journal.pone.0333472 (PMC12478895; doi:10.1371/journal.pone.0333472)
Supplement: S5 Table — (DOCX) [file pone.0333472.s005.docx]

**Supplementary Information**

**S5 Table. Sex interaction analysis.**

|  | **Associations between VAI and HOMA-IR** | | | **Associations between HOMA-IR and KDMAge** | | | **Associations between HOMA-IR and KDMAgeAccel risk** | | |
| --- | --- | --- | --- | --- | --- | --- | --- | --- | --- |
|  | **β (95% CI)** | ***P*-value** | ***P* for interaction** | **β (95% CI)** | ***P*-value** | ***P* for interaction** | **OR (95% CI)** | ***P*-value** | ***P* for interaction** |
| Females | 0.50 (0.33–0.66) | <0.001 | 0.308 | 0.35 (0.25–0.44) | <0.001 | 0.352 | 1.07 (1.05–1.10) | <0.001 | 0.584 |
| Males | 0.36 (0.24–0.48) | <0.001 |  | 0.34 (0.23–0.45) | <0.001 |  | 1.07 (1.05–1.10) | <0.001 |  |

The models were adjusted for age, race, education, marital status, poverty status, smoking status, alcohol consumption, M/VPA, HTN, CVD, cancer, and CKD.

VAI, visceral adiposity index; HOMA-IR, homeostasis model assessment of insulin resistance; KDMAge, Klemera-Doubal method age; KDMAgeAccel, KDMAge acceleration; CI, confidence interval; OR, odds ratio.
