# Supplementary material for: Sex differences in the association between visceral adiposity index and biological aging: A cross-sectional analysis of NHANES 1999–2018 with mediation by insulin resistance
Source: PLoS One. 2025 Sep 29;20(9):e0333472. doi: 10.1371/journal.pone.0333472 (PMC12478895; doi:10.1371/journal.pone.0333472)
Supplement: S6 Table — (DOCX) [file pone.0333472.s006.docx]

**Supplementary Information**

**S6 Table. Mediation analysis of HOMA-IR in the association between VAI and biological aging.**

|  | | **Whole Population** | | | **Females** | | | **Males** | | |
| --- | --- | --- | --- | --- | --- | --- | --- | --- | --- | --- |
|  |  | **β (95% CI)** | ***P*-value** | **Mediation Proportion (%)** | **β (95% CI)** | ***P*-value** | **Mediation Proportion (%)** | **β (95% CI)** | ***P*-value** | **Mediation Proportion (%)** |
| DKMAge | Indirect | 0.145 (0.091–0.159) | <0.001 | 18.92 | 0.139 (0.077–0.164) | <0.001 | 12.71 | 0.125 (0.071–0.161) | <0.001 | 21.67 |
|  | Direct | 0.623 (0.507–0.746) | <0.001 |  | 0.957 (0.722–1.100) | <0.001 |  | 0.452 (0.343–0.631) | <0.001 |  |
|  | Total | 0.768 (0.620–0.871) | <0.001 |  | 1.096 (0.831–1.227) | <0.001 |  | 0.578 (0.438–0.764) | <0.001 |  |
| DKMAgeAccel  risk | Indirect | 0.005 (0.003–0.006) | <0.001 | 20.90 | 0.004 (0.002–0.006) | <0.001 | 10.39 | 0.005 (0.002–0.006) | <0.001 | 27.09 |
|  | Direct | 0.019 (0.014–0.023) | <0.001 |  | 0.036 (0.023–0.037) | <0.001 |  | 0.012 (0.008–0.018) | <0.001 |  |
|  | Total | 0.024 (0.019–0.027) | <0.001 |  | 0.041 (0.028–0.041) | <0.001 |  | 0.017 (0.012–0.022) | <0.001 |  |

The models were adjusted for age, sex (only in the model of the whole population), race, education, marital status, poverty status, smoking status, alcohol consumption, M/VPA, HTN, CVD, cancer, and CKD. VAI, visceral adiposity index; HOMA-IR, homeostasis model assessment of insulin resistance; KDMAge, Klemera-Doubal method age; KDMAgeAccel, KDMAge acceleration; CI, confidence interval.
