# Supplementary material for: Sex differences in the association between visceral adiposity index and biological aging: A cross-sectional analysis of NHANES 1999–2018 with mediation by insulin resistance
Source: PLoS One. 2025 Sep 29;20(9):e0333472. doi: 10.1371/journal.pone.0333472 (PMC12478895; doi:10.1371/journal.pone.0333472)
Supplement: S7 Table — (DOCX) [file pone.0333472.s007.docx]

**Supplementary Information**

**S7 Table. Sex interaction analysis after additional adjustment for HOMA-IR.**

|  | **Associations between VAI and KDMAge** | | | **Associations between VAI and KDMAgeAccel risk** | | |
| --- | --- | --- | --- | --- | --- | --- |
|  | **β (95% CI)** | ***P*-value** | ***P* for interaction** | **OR (95% CI)** | ***P*-value** | ***P* for interaction** |
| Females | 0.91 (0.71–1.10) | <0.001 | 0.016 | 1.19 (1.13–1.25) | <0.001 | 0.005 |
| Males | 0.48 (0.33–0.62) | <0.001 |  | 1.07 (1.05–1.10) | <0.001 |  |

The models were adjusted for age, race, education, marital status, poverty status, smoking status, alcohol consumption, M/VPA, HTN, CVD, cancer, CKD, and HOMA-IR.

HOMA-IR, homeostasis model assessment of insulin resistance; VAI, visceral adiposity index; KDMAge, Klemera-Doubal method age; KDMAgeAccel, KDMAge acceleration; CI, confidence interval; OR, odds ratio.
