# Supplementary material for: Sex differences in the association between visceral adiposity index and biological aging: A cross-sectional analysis of NHANES 1999–2018 with mediation by insulin resistance
Source: PLoS One. 2025 Sep 29;20(9):e0333472. doi: 10.1371/journal.pone.0333472 (PMC12478895; doi:10.1371/journal.pone.0333472)
Supplement: S8 Table — (DOCX) [file pone.0333472.s008.docx]

**Supplementary Information**

**S8 Table. Sex interaction analysis after additional adjustment for DM and HDL.**

|  | **Associations between VAI and KDMAge** | | | **Associations between VAI and KDMAgeAccel risk** | | |
| --- | --- | --- | --- | --- | --- | --- |
|  | **β (95% CI)** | ***P*-value** | ***P* for interaction** | **OR (95% CI)** | ***P*-value** | ***P* for interaction** |
| Females | 1.15 (0.86–1.45) | <0.001 | 0.037 | 1.25 (1.18–1.32) | <0.001 | 0.008 |
| Males | 0.42 (0.27–0.56) | <0.001 |  | 1.07 (1.04–1.09) | <0.001 |  |

DM, diabetes mellitus; HDL, high-density lipoprotein; VAI, visceral adiposity index; KDMAge, Klemera-Doubal method age; KDMAgeAccel, KDMAge acceleration; CI, confidence interval; OR, odds ratio. The models were adjusted for age, race, education, marital status, poverty status, smoking status, alcohol consumption, M/VPA, HTN, CVD, cancer, CKD, DM and HDL.
