# Supplementary material for: Sex differences in the association between visceral adiposity index and biological aging: A cross-sectional analysis of NHANES 1999–2018 with mediation by insulin resistance
Source: PLoS One. 2025 Sep 29;20(9):e0333472. doi: 10.1371/journal.pone.0333472 (PMC12478895; doi:10.1371/journal.pone.0333472)
Supplement: S9 Table — (DOCX) [file pone.0333472.s009.docx]

**Supplementary Information**

**S9 Table. Multivariate regression analysis after additional adjustment for DM and HDL.**

|  | **Associations between VAI and KDMAge** | | **Associations between VAI and KDMAgeAccel risk** | |
| --- | --- | --- | --- | --- |
|  | **β (95% CI)** | ***P***-value | **OR (95% CI)** | ***P***-value |
| Whole pupulation |  | | | |
| VAI continue | 0.71 (0.57–0.85) | <0.001 | 1.12 (1.09–1.15) | <0.001 |
| VAI quantile |  | | | |
| Q1 | 0.00 (Reference) |  | 1.00 (Reference) |  |
| Q2 | 2.61 (1.96–3.25) | <0.001 | 1.37 (1.19–1.59) | <0.001 |
| Q3 | 5.43 (4.61–6.26) | <0.001 | 2.05 (1.73–2.43) | <0.001 |
| Q4 | 8.93 (7.99–9.87) | <0.001 | 3.18 (2.67–3.80) | <0.001 |
| *P*-trend |  | <0.001 |  | <0.001 |
| Females |  | | | |
| VAI continue | 1.15 (0.86–1.45) | <0.001 | 1.25 (1.18–1.32) | <0.001 |
| VAI quantile |  | | | |
| Q1 | 0.00 (Reference) |  | 1.00 (Reference) |  |
| Q2 | 3.50 (2.77–4.24) | <0.001 | 1.62 (1.33–1.96) | <0.001 |
| Q3 | 7.32 (6.34–8.30) | <0.001 | 2.98 (2.40–3.71) | <0.001 |
| Q4 | 12.30 (11.19–13.41) | <0.001 | 5.70 (4.53–7.17) | <0.001 |
| *P*-trend |  | <0.001 |  | <0.001 |
| Males |  | | | |
| VAI continue | 0.42 (0.27–0.56) | <0.001 | 1.07 (1.04–1.09) | <0.001 |
| VAI quantile |  | | | |
| Q1 | 0.00 (Reference) |  | 1.00 (Reference) |  |
| Q2 | 1.87 (0.91–2.82) | <0.001 | 1.26 (1.04–1.52) | 0.02 |
| Q3 | 3.55 (2.18–4.91) | <0.001 | 1.52 (1.19–1.94) | <0.001 |
| Q4 | 6.28 (4.80–7.76) | <0.001 | 2.16 (1.66–2.80) | <0.001 |
| *P*-trend |  | <0.001 |  | <0.001 |

The models were adjusted for age, sex (only in the model of the whole population), race, education, marital status, poverty status, smoking status, alcohol consumption, M/VPA, HTN, CVD, cancer, CKD, DM and HDL. DM, diabetes mellitus; HDL, high-density lipoprotein; VAI, visceral adiposity index; KDMAge, Klemera-Doubal method age; KDMAgeAccel, KDMAge acceleration; CI, confidence interval; OR, odds ratio.
