# Supplementary material for: Sex differences in the association between visceral adiposity index and biological aging: A cross-sectional analysis of NHANES 1999–2018 with mediation by insulin resistance
Source: PLoS One. 2025 Sep 29;20(9):e0333472. doi: 10.1371/journal.pone.0333472 (PMC12478895; doi:10.1371/journal.pone.0333472)
Supplement: S10 Table — (DOCX) [file pone.0333472.s010.docx]

**Supplementary Information**

**S10 Table. Threshold effect analysis after additional adjustment for DM and HDL.**

|  | **Associations between VAI and KDMAge** | | **Associations between VAI and KDMAgeAccel risk** | |
| --- | --- | --- | --- | --- |
|  | **β (95% CI)** | ***P*-value** | **OR (95% CI)** | ***P*-value** |
| Whole population |  | | | |
| Standard linear regression | 0.66 (0.57–0.74) | <0.001 | 1.10 (1.08–1.11) | <0.001 |
| Two-piecewise linear regression |  | | | |
| K | 2.601 |  | 2.453 |  |
| <K | 4.01 (3.55–4.47) | <0.001 | 1.74 (1.60–1.90) | <0.001 |
| ≥K | 0.35 (0.24–0.46) | <0.001 | 1.05 (1.03–1.06) | <0.001 |
| Likelihood ratio |  | <0.001 |  | <0.001 |
| Females |  | | | |
| Standard linear regression | 1.03 (0.91–1.15) | <0.001 | 1.18 (1.15–1.22) | <0.001 |
| Two-piecewise linear regression |  | | | |
| K | 2.845 |  | 2.285 |  |
| <K | 5.39 (4.87–5.90) | <0.001 | 2.67 (2.32–3.08) | <0.001 |
| ≥K | 0.43 (0.27–0.60) | <0.001 | 1.07 (1.04–1.11) | <0.001 |
| Likelihood ratio |  | <0.001 |  | <0.001 |
| Males |  | | | |
| Standard linear regression | 0.42 (0.29–0.54) | <0.001 | 1.06 (1.04–1.08) | <0.001 |
| Two-piecewise linear regression |  | | | |
| K | 2.529 |  | 2.502 |  |
| <K | 2.73 (1.98–3.48) | <0.001 | 1.35 (1.19–1.52) | <0.001 |
| ≥K | 0.25 (0.10–0.41) | 0.001 | 1.04 (1.01–1.06) | 0.001 |
| Likelihood ratio |  | <0.001 |  | <0.001 |

The models were adjusted for age, sex (only in the model of the whole population), race, education, marital status, poverty status, smoking status, alcohol consumption, M/VPA, HTN, CVD, cancer, CKD, DM and HDL. DM, diabetes mellitus; HDL, high-density lipoprotein; VAI, visceral adiposity index; KDMAge, Klemera-Doubal method age; KDMAgeAccel, KDMAge acceleration; CI, confidence interval; OR, odds ratio.
