# Supplementary material for: Sex differences in the association between visceral adiposity index and biological aging: A cross-sectional analysis of NHANES 1999–2018 with mediation by insulin resistance
Source: PLoS One. 2025 Sep 29;20(9):e0333472. doi: 10.1371/journal.pone.0333472 (PMC12478895; doi:10.1371/journal.pone.0333472)
Supplement: S11 Table — (DOCX) [file pone.0333472.s011.docx]

**Supplementary Information**

**S11 Table. Mediation analysis after additional adjustment for DM and HDL.**

|  | | **Whole Population** | | | **Females** | | | **Males** | | |
| --- | --- | --- | --- | --- | --- | --- | --- | --- | --- | --- |
|  |  | **β (95% CI)** | ***P*-value** | **Mediation Proportion (%)** | **β (95% CI)** | ***P*-value** | **Mediation Proportion (%)** | **β (95% CI)** | ***P*-value** | **Mediation Proportion (%)** |
| DKMAge | Indirect | 0.058 (0.033–0.073) | <0.001 | 8.24 | 0.071 (0.031–0.092) | <0.001 | 5.80 | 0.041 (0.020–0.064) | <0.001 | 10.54 |
|  | Direct | 0.650 (0.536–0.807) | <0.001 |  | 1.158 (0.864–1.386) | <0.001 |  | 0.351 (0.245–0.539) | <0.001 |  |
|  | Total | 0.708 (0.578–0.866) | <0.001 |  | 1.230 (0.911–1.441) | <0.001 |  | 0.393 (0.280–0.581) | <0.001 |  |
| DKMAgeAccel  risk | Indirect | 0.002 (0.001–0.003) | <0.001 | 10.62 | 0.003 (0.001–0.004) | <0.001 | 5.42 | 0.002 (0.001–0.003) | <0.001 | 14.46 |
|  | Direct | 0.020 (0.014–0.023) | <0.001 |  | 0.045 (0.027–0.045) | <0.001 |  | 0.010 (0.006–0.015) | <0.001 |  |
|  | Total | 0.022 (0.015–0.025) | <0.001 |  | 0.047 (0.029–0.047) | <0.001 |  | 0.012 (0.008–0.017) | <0.001 |  |

The models were adjusted for age, sex (only in the model of the whole population), race, education, marital status, poverty status, smoking status, alcohol consumption, M/VPA, HTN, CVD, cancer, CKD, DM and HDL. KDMAge, Klemera-Doubal method age; KDMAgeAccel, KDMAge acceleration; CI, confidence interval.
