# Supplementary material for: Sex differences in the association between visceral adiposity index and biological aging: A cross-sectional analysis of NHANES 1999–2018 with mediation by insulin resistance
Source: PLoS One. 2025 Sep 29;20(9):e0333472. doi: 10.1371/journal.pone.0333472 (PMC12478895; doi:10.1371/journal.pone.0333472)
Supplement: S12 Table — (DOCX) [file pone.0333472.s012.docx]

**Supplementary Information**

**S12 Table. Sex interaction analysis following exclusion of DM participants.**

|  | **Associations between VAI and KDMAge** | | | **Associations between VAI and KDMAgeAccel risk** | | |
| --- | --- | --- | --- | --- | --- | --- |
|  | **β (95% CI)** | ***P*-value** | ***P* for interaction** | **OR (95% CI)** | ***P*-value** | ***P* for interaction** |
| Females | 1.13 (0.81 ~ 1.46) | <0.001 | 0.007 | 1.27 (1.21 ~ 1.34) | <0.001 | <0.001 |
| Males | 0.59 (0.43 ~ 0.75) | <0.001 |  | 1.10 (1.06 ~ 1.14) | <0.001 |  |

DM, diabetes mellitus; VAI, visceral adiposity index; KDMAge, Klemera-Doubal method age; KDMAgeAccel, KDMAge acceleration; CI, confidence interval; OR, odds ratio. The models were adjusted for age, race, education, marital status, poverty status, smoking status, alcohol consumption, M/VPA, HTN, CVD, cancer, and CKD.
