# Supplementary material for: Sex differences in the association between visceral adiposity index and biological aging: A cross-sectional analysis of NHANES 1999–2018 with mediation by insulin resistance
Source: PLoS One. 2025 Sep 29;20(9):e0333472. doi: 10.1371/journal.pone.0333472 (PMC12478895; doi:10.1371/journal.pone.0333472)
Supplement: S13 Table — (DOCX) [file pone.0333472.s013.docx]

**Supplementary Information**

**S13 Table. Multivariate regression analysis following exclusion of DM participants.**

|  | **Associations between VAI and KDMAge** | | **Associations between VAI and KDMAgeAccel risk** | |
| --- | --- | --- | --- | --- |
|  | **β (95% CI)** | ***P***-value | **OR (95% CI)** | ***P***-value |
| Whole pupulation |  | | | |
| VAI continue | 0.77 (0.62–0.93) | <0.001 | 1.15 (1.12–1.18) | <0.001 |
| VAI quantile |  | | | |
| Q1 | 0.00 (Reference) |  | 1.00 (Reference) |  |
| Q2 | 1.47 (0.82–2.11) | <0.001 | 1.34 (1.18–1.53) | <0.001 |
| Q3 | 3.44 (2.69–4.20) | <0.001 | 1.77 (1.50–2.09) | <0.001 |
| Q4 | 6.11 (5.30–6.92) | <0.001 | 2.70 (2.33–3.13) | <0.001 |
| *P*-trend |  | <0.001 |  | <0.001 |
| Females |  | | | |
| VAI continue | 1.13 (0.81–1.46) | <0.001 | 1.27 (1.21–1.34) | <0.001 |
| VAI quantile |  | | | |
| Q1 | 0.00 (Reference) |  | 1.00 (Reference) |  |
| Q2 | 1.53 (0.80–2.27) | <0.001 | 1.36 (1.13–1.65) | 0.002 |
| Q3 | 3.76 (2.90–4.62) | <0.001 | 2.07 (1.67–2.55) | <0.001 |
| Q4 | 6.97 (5.97–7.96) | <0.001 | 3.46 (2.80–4.27) | <0.001 |
| *P*-trend |  | <0.001 |  | <0.001 |
| Males |  | | | |
| VAI continue | 0.59 (0.43–0.75) | <0.001 | 1.10 (1.06–1.14) | <0.001 |
| VAI quantile |  | | | |
| Q1 | 0.00 (Reference) |  | 1.00 (Reference) |  |
| Q2 | 2.08 (1.05–3.10) | <0.001 | 1.46 (1.19–1.79) | <0.001 |
| Q3 | 3.55 (2.41–4.70) | <0.001 | 1.61 (1.27–2.04) | <0.001 |
| Q4 | 5.97 (4.77–7.17) | <0.001 | 2.43 (1.96–3.02) | <0.001 |
| *P*-trend |  | <0.001 |  | <0.001 |

The models were adjusted for age, sex (only in the model of the whole population), race, education, marital status, poverty status, smoking status, alcohol consumption, M/VPA, HTN, CVD, cancer, and CKD. DM, diabetes mellitus; VAI, visceral adiposity index; KDMAge, Klemera-Doubal method age; KDMAgeAccel, KDMAge acceleration; CI, confidence interval; OR, odds ratio.
