# Supplementary material for: Sex differences in the association between visceral adiposity index and biological aging: A cross-sectional analysis of NHANES 1999–2018 with mediation by insulin resistance
Source: PLoS One. 2025 Sep 29;20(9):e0333472. doi: 10.1371/journal.pone.0333472 (PMC12478895; doi:10.1371/journal.pone.0333472)
Supplement: S14 Table — (DOCX) [file pone.0333472.s014.docx]

**Supplementary Information**

**S14 Table. Threshold effect analysis following exclusion of DM participants.**

|  | **Associations between VAI and KDMAge** | | **Associations between VAI and KDMAgeAccel risk** | |
| --- | --- | --- | --- | --- |
|  | **β (95% CI)** | ***P*-value** | **OR (95% CI)** | ***P*-value** |
| Whole population |  | | | |
| Standard linear regression | 0.71 (0.62–0.80) | <0.001 | 1.12 (1.10–1.15) | <0.001 |
| Two-piecewise linear regression |  | | | |
| K | 2.845 |  | 2.543 |  |
| <K | 2.34 (2.00–2.68) | <0.001 | 1.50 (1.39–1.62) | <0.001 |
| ≥K | 0.24 (0.12–0.36) | <0.001 | 1.04 (1.02–1.06) | <0.001 |
| Likelihood ratio |  | <0.001 |  | <0.001 |
| Females |  | | | |
| Standard linear regression | 0.96 (0.83–1.09) | <0.001 | 1.20 (1.16–1.24) | <0.001 |
| Two-piecewise linear regression |  | | | |
| K | 3.413 |  | 0.603 |  |
| <K | 2.48 (2.12–2.84) | <0.001 | 1.07 (0.20–5.66) | 0.941 |
| ≥K | 0.18 (0.02–0.39) | 0.004 | 1.18 (1.14–1.22) | <0.001 |
| Likelihood ratio |  | <0.001 |  | <0.001 |
| Males |  | | | |
| Standard linear regression | 0.59 (0.46–0.71) | <0.001 | 1.09 (1.07–1.12) | <0.001 |
| Two-piecewise linear regression |  | | | |
| K | 2.512 |  | 2.528 |  |
| <K | 2.35 (1.73–2.96) | <0.001 | 1.35 (1.22–1.51) | <0.001 |
| ≥K | 0.24 (0.08–0.39) | 0.003 | 1.04 (1.02–1.07) | 0.002 |
| Likelihood ratio |  | <0.001 |  | <0.001 |

The models were adjusted for age, sex (only in the model of the whole population), race, education, marital status, poverty status, smoking status, alcohol consumption, M/VPA, HTN, CVD, cancer, and CKD. DM, diabetes mellitus; VAI, visceral adiposity index; KDMAge, Klemera-Doubal method age; KDMAgeAccel, KDMAge acceleration; CI, confidence interval; OR, odds ratio.
