# Supplementary material for: Sex differences in the association between visceral adiposity index and biological aging: A cross-sectional analysis of NHANES 1999–2018 with mediation by insulin resistance
Source: PLoS One. 2025 Sep 29;20(9):e0333472. doi: 10.1371/journal.pone.0333472 (PMC12478895; doi:10.1371/journal.pone.0333472)
Supplement: S15 Table — (DOCX) [file pone.0333472.s015.docx]

**Supplementary Information**

**S15 Table. Mediation analysis following exclusion of DM participants.**

|  | | **Whole Population** | | | **Females** | | | **Males** | | |
| --- | --- | --- | --- | --- | --- | --- | --- | --- | --- | --- |
|  |  | **β (95% CI)** | ***P*-value** | **Mediation Proportion (%)** | **β (95% CI)** | ***P*-value** | **Mediation Proportion (%)** | **β (95% CI)** | ***P*-value** | **Mediation Proportion (%)** |
| DKMAge | Indirect | 0.146 (0.123–0.191) | <0.001 | 17.85 | 0.156 (0.101–0.203) | <0.001 | 11.11 | 0.139 (0.111–0.203) | <0.001 | 21.56 |
|  | Direct | 0.674 (0.501–0.767) | <0.001 |  | 1.247 (0.754–1.250) | <0.001 |  | 0.504 (0.302–0.594) | <0.001 |  |
|  | Total | 0.820 (0.639–0.938) | <0.001 |  | 1.403 (0.876–1.415) | <0.001 |  | 0.643 (0.436–0.768) | <0.001 |  |
| DKMAgeAccel  risk | Indirect | 0.006 (0.005–0.007) | <0.001 | 22.59 | 0.007 (0.004–0.009) | <0.001 | 18.98 | 0.005 (0.004–0.007) | <0.001 | 29.46 |
|  | Direct | 0.020 (0.012–0.021) | <0.001 |  | 0.032 (0.024–0.039) | <0.001 |  | 0.013 (0.006–0.015) | <0.001 |  |
|  | Total | 0.026 (0.018–0.027) | <0.001 |  | 0.039 (0.031–0.045) | <0.001 |  | 0.018 (0.012–0.021) | <0.001 |  |

The models were adjusted for age, sex (only in the model of the whole population), race, education, marital status, poverty status, smoking status, alcohol consumption, M/VPA, HTN, CVD, cancer, and CKD. DM, diabetes mellitus; KDMAge, Klemera-Doubal method age; KDMAgeAccel, KDMAge acceleration; CI, confidence interval.
