# Supplementary material for: Sex differences in the association between visceral adiposity index and biological aging: A cross-sectional analysis of NHANES 1999–2018 with mediation by insulin resistance
Source: PLoS One. 2025 Sep 29;20(9):e0333472. doi: 10.1371/journal.pone.0333472 (PMC12478895; doi:10.1371/journal.pone.0333472)
Supplement: S16 Table — (DOCX) [file pone.0333472.s016.docx]

**Supplementary Information**

**S16 Table. Subgroup analyses of VAI–HOMA-IR associations.**

| Race/Ethnicity | N (%) | **VAI–HOMA-IR associations** | | ***P*** for interaction |
| --- | --- | --- | --- | --- |
|  |  | **β (95% CI)** | ***P***-value |  |
| Whole population |  | | | |
| Mexican American | 3357 (17.23) | 0.47 (0.31–0.63) | <0.001 | 0.044 |
| Non-Hispanic White | 8996 (46.17) | 0.40 (0.30–0.50) | <0.001 |  |
| Non-Hispanic Black | 3803 (19.52) | 1.15 (0.77–1.52) | <0.001 |  |
| Other | 3330 (17.09) | 0.33 (0.05–0.61) | 0.023 |  |
| Females |  | | | |
| Mexican American | 1641 (16.86) | 0.54 (0.30–0.78) | <0.001 | 0.018 |
| Non-Hispanic White | 4420 (45.42) | 0.45 (0.27–0.64) | <0.001 |  |
| Non-Hispanic Black | 1966 (20.20) | 1.33 (0.76–1.90) | <0.001 |  |
| Other | 1705 (17.52) | 0.53 (0.24–0.81) | <0.001 |  |
| Males |  | | | |
| Mexican American | 1716 (17.59) | 0.41 (0.18–0.64) | <0.001 | 0.204 |
| Non-Hispanic White | 4576 (46.91) | 0.36 (0.25–0.47) | <0.001 |  |
| Non-Hispanic Black | 1837 (18.83) | 1.01 (0.55–1.47) | <0.001 |  |
| Other | 1625 (16.66) | 0.23 (-0.06–0.52) | 0.118 |  |

The models were adjusted for age, sex (only in the model of the whole population), education, marital status, poverty status, smoking status, alcohol consumption, M/VPA, HTN, CVD, cancer, and CKD. HOMA-IR, homeostasis model assessment of insulin resistance; VAI, visceral adiposity index; CI, confidence interval.
