# Supplementary material for: Sex differences in the association between visceral adiposity index and biological aging: A cross-sectional analysis of NHANES 1999–2018 with mediation by insulin resistance
Source: PLoS One. 2025 Sep 29;20(9):e0333472. doi: 10.1371/journal.pone.0333472 (PMC12478895; doi:10.1371/journal.pone.0333472)
Supplement: S17 Table — (DOCX) [file pone.0333472.s017.docx]

**Supplementary Information**

**S17 Table. Subgroup analyses of HOMA-IR–BA associations.**

| Race/Ethnicity | N (%) | **HOMA-IR–KDMAge associations** | | ***P* for interaction** | **HOMA-IR–KDMAgeAccel associations** | | ***P* for interaction** |
| --- | --- | --- | --- | --- | --- | --- | --- |
|  |  | **β (95% CI)** | ***P*-value** |  | **OR (95% CI)** | ***P*-value** |  |
| Whole population |  | | | | | | |
| Mexican American | 3357 (17.23) | 0.20 (0.01–0.42) | 0.007 | 0.481 | 1.05 (1.01–1.11) | 0.011 | 0.609 |
| Non-Hispanic White | 8996 (46.17) | 0.37 (0.28–0.47) | <0.001 |  | 1.08 (1.06–1.11) | <0.001 |  |
| Non-Hispanic Black | 3803 (19.52) | 0.34 (0.22–0.46) | <0.001 |  | 1.05 (1.02–1.08) | 0.001 |  |
| Other | 3330 (17.09) | 0.38 (0.17–0.58) | <0.001 |  | 1.07 (1.03–1.12) | 0.001 |  |
| Females |  | | | | | | |
| Mexican American | 1641 (16.86) | 0.19 (-0.03–0.41) | 0.091 | 0.479 | 1.06 (1.01–1.11) | 0.016 | 0.816 |
| Non-Hispanic White | 4420 (45.42) | 0.37 (0.23–0.51) | <0.001 |  | 1.08 (1.04–1.12) | <0.001 |  |
| Non-Hispanic Black | 1966 (20.20) | 0.30 (0.16–0.44) | <0.001 |  | 1.05 (1.01–1.09) | 0.011 |  |
| Other | 1705 (17.52) | 0.42 (0.21–0.62) | <0.001 |  | 1.07 (1.02–1.13) | 0.013 |  |
| Males |  | | | | | | |
| Mexican American | 1716 (17.59) | 0.19 (-0.11–0.49) | 0.219 | 0.560 | 1.04 (0.95–1.13) | 0.407 | 0.732 |
| Non-Hispanic White | 4576 (46.91) | 0.37 (0.24–0.50) | <0.001 |  | 1.08 (1.05–1.11) | <0.001 |  |
| Non-Hispanic Black | 1837 (18.83) | 0.38 (0.17–0.59) | <0.001 |  | 1.05 (1.00–1.10) | 0.06 |  |
| Other | 1625 (16.66) | 0.35 (0.13–0.57) | 0.002 |  | 1.08 (1.02–1.14) | 0.011 |  |

The models were adjusted for age, sex (only in the model of the whole population), education, marital status, poverty status, smoking status, alcohol consumption, M/VPA, HTN, CVD, cancer, and CKD. HOMA-IR, homeostasis model assessment of insulin resistance; KDMAge, Klemera-Doubal method age; KDMAgeAccel, KDMAge acceleration; CI, confidence interval.
