# Supplementary material for: Sex differences in the association between visceral adiposity index and biological aging: A cross-sectional analysis of NHANES 1999–2018 with mediation by insulin resistance
Source: PLoS One. 2025 Sep 29;20(9):e0333472. doi: 10.1371/journal.pone.0333472 (PMC12478895; doi:10.1371/journal.pone.0333472)
Supplement: S18 Table — (DOCX) [file pone.0333472.s018.docx]

**Supplementary Information**

**S18 Table. Subgroup analyses of VAI–BA associations for different race.**

| **Race/Ethnicity** | **N (%)** | **VAI–KDMAge associations** | | ***P* for interaction** | **VAI–KDMAgeAccel associations** | | ***P* for interaction** |
| --- | --- | --- | --- | --- | --- | --- | --- |
|  |  | **β (95% CI)** | ***P*-value** |  | **OR (95% CI)** | ***P*-value** |  |
| Whole population |  | | | | | | |
| Mexican American | 3357 (17.23) | 0.64 (0.40–0.88) | <0.001 | 0.021 | 1.08 (1.03–1.13) | 0.002 | 0.006 |
| Non-Hispanic White | 8996 (46.17) | 0.85 (0.68–1.01) | <0.001 |  | 1.16 (1.13–1.20) | <0.001 |  |
| Non-Hispanic Black | 3803 (19.52) | 1.15 (0.64–1.66) | <0.001 |  | 1.15 (1.06–1.25) | 0.001 |  |
| Other | 3330 (17.09) | 0.43 (0.15–0.71) | 0.003 |  | 1.08 (1.03–1.12) | <0.001 |  |
| Females |  | | | | | | |
| Mexican American | 1641 (16.86) | 0.77 (0.42–1.13) | <0.001 | 0.334 | 1.18 (1.08–1.28) | <0.001 | 0.642 |
| Non-Hispanic White | 4420 (45.42) | 1.04 (0.78–1.30) | <0.001 |  | 1.24 (1.18–1.31) | <0.001 |  |
| Non-Hispanic Black | 1966 (20.20) | 1.48 (0.88–2.08) | <0.001 |  | 1.22 (1.10–1.36) | <0.001 |  |
| Other | 1705 (17.52) | 0.92 (0.42–1.41) | <0.001 |  | 1.14 (1.06–1.23) | <0.001 |  |
| Males |  | | | | | | |
| Mexican American | 1716 (17.59) | 0.53 (0.22–0.84) | 0.001 | 0.025 | 1.04 (0.99–1.09) | 0.141 | 0.026 |
| Non-Hispanic White | 4576 (46.91) | 0.73 (0.50–0.95) | <0.001 |  | 1.13 (1.08–1.18) | <0.001 |  |
| Non-Hispanic Black | 1837 (18.83) | 0.84 (0.13–1.56) | 0.023 |  | 1.09 (0.99–1.20) | 0.095 |  |
| Other | 1625 (16.66) | 0.27 (0.01–0.52) | 0.041 |  | 1.06 (1.02–1.11) | 0.005 |  |

The models were adjusted for age, sex (only in the model of the whole population), education, marital status, poverty status, smoking status, alcohol consumption, M/VPA, HTN, CVD, cancer, and CKD. VAI, visceral adiposity index; KDMAge, Klemera-Doubal method age; KDMAgeAccel, KDMAge acceleration; CI, confidence interval.
