# Supplementary material for: Sex differences in the association between visceral adiposity index and biological aging: A cross-sectional analysis of NHANES 1999–2018 with mediation by insulin resistance
Source: PLoS One. 2025 Sep 29;20(9):e0333472. doi: 10.1371/journal.pone.0333472 (PMC12478895; doi:10.1371/journal.pone.0333472)
Supplement: S19 Table — (DOCX) [file pone.0333472.s019.docx]

**Supplementary Information**

**S19 Table. Subgroup analyses of VAI–BA associations for different age groups.**

| **Age groups (years)** | **N (%)** | **VAI–KDMAge associations** | | ***P* for interaction** | **VAI–KDMAgeAccel associations** | | ***P* for interaction** |
| --- | --- | --- | --- | --- | --- | --- | --- |
|  |  | **β (95% CI)** | ***P*-value** |  | **OR (95% CI)** | ***P*-value** |  |
| Whole population |  | | | | | | |
| 20–39 | 6358 (32.63) | 1.05 (0.83–1.28) | <0.001 | 0.017 | 1.22 (1.16–1.29) | <0.001 | 0.039 |
| 40–59 | 6527 (33.50) | 0.64 (0.49–0.79) | <0.001 |  | 1.11 (1.07–1.16) | <0.001 |  |
| ≥ 60 | 6601 (33.88) | 0.85 (0.56–1.14) | <0.001 |  | 1.15 (1.09–1.21) | <0.001 |  |
| Females |  | | | | | | |
| 20–39 | 3148 (32.35) | 1.29 (0.91–1.67) | <0.001 | 0.548 | 1.40 (1.27–1.54) | <0.001 | 0.209 |
| 40–59 | 3298 (33.89) | 0.93 (0.60–1.25) | <0.001 |  | 1.18 (1.10–1.27) | <0.001 |  |
| ≥ 60 | 3286 (33.76) | 1.01 (0.69–1.33) | <0.001 |  | 1.19 (1.12–1.26) | <0.001 |  |
| Males |  | | | | | | |
| 20–39 | 3210 (32.91) | 0.91 (0.60–1.21) | <0.001 | 0.052 | 1.15 (1.08–1.22) | <0.001 | 0.335 |
| 40–59 | 3229 (33.10) | 0.46 (0.31–0.61) | <0.001 |  | 1.08 (1.04–1.13) | <0.001 |  |
| ≥ 60 | 3315 (33.99) | 0.71 (0.18–1.25) | 0.010 |  | 1.10 (1.02–1.19) | 0.013 |  |

The models were adjusted for sex (only in the model of the whole population), race, education, marital status, poverty status, smoking status, alcohol consumption, M/VPA, HTN, CVD, cancer, and CKD. VAI, visceral adiposity index; KDMAge, Klemera-Doubal method age; KDMAgeAccel, KDMAge acceleration; CI, confidence interval.
