# Supplementary material for: Sex differences in the association between visceral adiposity index and biological aging: A cross-sectional analysis of NHANES 1999–2018 with mediation by insulin resistance
Source: PLoS One. 2025 Sep 29;20(9):e0333472. doi: 10.1371/journal.pone.0333472 (PMC12478895; doi:10.1371/journal.pone.0333472)
Supplement: S2 Fig — (DOCX) [file pone.0333472.s021.docx]

**Supplementary Information**


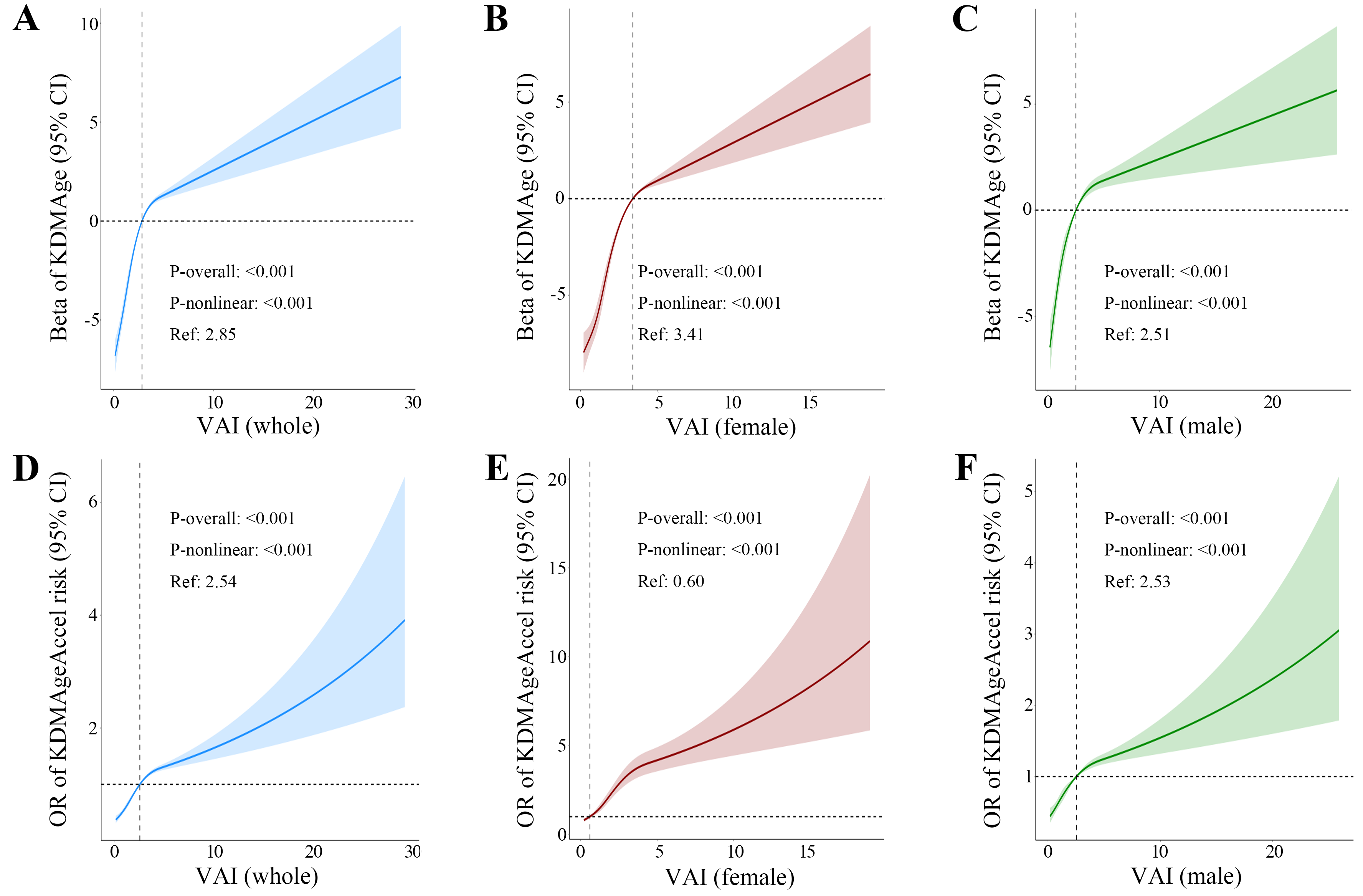


**S2 Fig. Restricted cubic splines analyses following exclusion of DM participants.** Associations between VAI and KDMAge/KDMAgeAccel risk among the whole population (**A, D**), the females (**B, E**), and the males (**C, F**). The calculation of Beta and OR in the figures is performed subsequent to adjustment for age, sex (only in the model of whole population), race, education, marital status, poverty status, smoking status, alcohol consumption, M/VPA, HTN, CVD, cancer, and CKD. DM, diabetes mellitus; VAI, visceral adiposity index; KDMAge, this is the biological age that is determined according to the Klemera-Doubal method; KDMAgeAccel, KDMAge acceleration; OR, odds ratio; Cl, confidence interval.
