# Supplementary material for: Sex differences in the association between visceral adiposity index and biological aging: A cross-sectional analysis of NHANES 1999–2018 with mediation by insulin resistance
Source: PLoS One. 2025 Sep 29;20(9):e0333472. doi: 10.1371/journal.pone.0333472 (PMC12478895; doi:10.1371/journal.pone.0333472)
Supplement: Attachment — Submitted filename: The original article entitled.docx [file pone.0333472.s023.docx]

The original article entitled “Sex differences in the association between visceral adiposity index and biological aging: a mediation analysis of insulin resistance” was well-written and easy to understand. The study investigated the association between visceral adiposity index and biological aging, with insulin resistance playing a mediating role in homeostasis between sexes. The sample size was large, and the authors made efforts to provide guidelines for obesity and aging. However, I have some suggestion to improve the paper.

The introduction was well-written, but too long. I suggest summarizing it.

The resolution of the Figures is low

It would be better to define clinical features, including HTN, DM, CKD, CVD and Cancer.
