# Supplementary material for: Sex differences in the association between visceral adiposity index and biological aging: A cross-sectional analysis of NHANES 1999–2018 with mediation by insulin resistance
Source: PLoS One. 2025 Sep 29;20(9):e0333472. doi: 10.1371/journal.pone.0333472 (PMC12478895; doi:10.1371/journal.pone.0333472)
Supplement: Attachment — Submitted filename: Response to Reviewers.docx [file pone.0333472.s024.docx]

# Response Letter

Dear PLOS ONE Editorial Team,

Thank you for giving us the opportunity to submit a revised draft of our manuscript titled “Sex differences in the association between visceral adiposity index and biological aging: a mediation analysis of insulin resistance” [PONE-D-25-26841R1] to ***PLOS One****.* We appreciate the time and effort that you and the reviewers have dedicated to providing valuable feedback on our manuscript. We are grateful to the reviewers for their insightful comments, which have improved the paper. We have incorporated changes that reflect all the suggestions and concerns provided by the reviewers. The reviewer comments are laid out below in *italicized font* and specific concerns have been numbered. Our replies appear in blue font and key points are emphasized in red. All corresponding revisions in the manuscript are marked in yellow. Furthermore, we would like to show the details as follows:

**Responses to Comments of Reviewer #1:**

*Comments 1: Add in the title the target population: Cross sectional analysis of NHANES (1999-2018) data.*

Response: We think this is an excellent suggestion. We have added the target population and database information to the title as suggested. The revised title now reads: "Sex differences in the association between visceral adiposity index and biological aging: a cross-sectional analysis of NHANES 1999–2018 with mediation by insulin resistance"

*Comments 2: Why did you use VAI rather than NHANES DXA data on Visceral adipose tissue “VAT” quantity (mass, volume, or surface area) as a more direct measure of visceral adiposity?*

Response: We sincerely appreciate the reviewer's insightful query regarding our selection of the visceral adiposity index (VAI) over DXA-derived visceral adipose tissue (VAT) quantification. We acknowledge the well-established value of VAT as a more direct measure of visceral adiposity, evidenced by its documented associations with diabetes mellitus [1], systemic inflammation [2], and cognitive function [3]. However, our methodological choice of VAI was based on three key considerations:

- Clinical applicability and feasibility

VAI integrates routinely measured clinical parameters (waist circumference, BMI, triglycerides, HDL) into a sex-specific formula. This allows broader implementation in resource-limited settings where DXA is unavailable, aligning with our goal of developing accessible biomarkers for public health use [4]. DXA, while providing precise anatomical VAT quantification, requires specialized equipment and expertise, limiting its scalability in population-based screening.

- Comprehensive pathophysiological assessment

Unlike VAT mass/volume/area, which solely reflects morphological adiposity, VAI simultaneously captures both visceral fat accumulation and associated metabolic dysfunction (dyslipidemia, insulin resistance) [5]. This dual capacity is critical because metabolic dysregulation, not fat mass alone, drives obesity-related aging mechanisms [6,7]. Our mediation results further validate VAI’s ability to reflect IR-mediated biological aging pathways.

- Data availability and cohort integrity

It is important to note that DXA-derived VAT data in NHANES were systematically collected from 2011 onward. Restricting analyses to DXA-available cycles (2011–2018) would reduce the sample size by more than 60% and disproportionately exclude older adults (who are underrepresented in DXA subsamples due to the exclusion criteria of 60 years or older). This would compromise the statistical power of sex-stratified mediation analyses and introduce selection bias. VAI facilitated the optimal utilization of 20-year nationally representative data (1999–2018).

To further address your concerns about VAT, we performed the following analyses using direct measures of VAT, including mass, volume, and area, to further validate the association between visceral adiposity and biological aging (KDMAge and KDMAgeAccel risk).

The results are consistent with our primary results concerning the VAI–biological aging association. This consistency suggests that both VAT and VAI serve as outstanding indicators for evaluating the link between visceral adiposity and advanced biological aging. Nevertheless, based on the three key considerations outlined previously, we maintained VAI as the primary measure of visceral adiposity for this study. We would like to once again express our gratitude to the reviewer for the attention to this important methodological dimension, which indeed highlights valuable directions for future research in this field.

The specific analysis process is as follows: Following full adjustment for covariates, multivariable linear and logistic regression analyses demonstrated significant positive correlations between VAT and measures of biological aging (Table 1). Furthermore, restricted cubic splines revealed significant nonlinear relationships between VAT and biological aging (Fig. 1).

**Table 1. Multivariable regression analyses.**

|  | **Associations between VAT and KDMAge** | | **Associations between VAT and KDMAgeAccel risk** | |
| --- | --- | --- | --- | --- |
|  | **β (95% CI)** | ***P*-value** | **OR (95% CI)** | ***P*-value** |
| **VAT mass** |  | | | |
| Continue | 0.014 (0.012, 0.016) | <0.001 | 1.001 (1.001, 1.002) | <0.001 |
| Quantile |  | | | |
| Q1 | 0.00 (Reference) |  | 1.00 (Reference) |  |
| Q2 | 3.269 (2.317, 4.220) | <0.001 | 1.339 (1.104, 1.623) | 0.004 |
| Q3 | 6.563 (5.688, 7.437) | <0.001 | 1.995 (1.615, 2.463) | <0.001 |
| Q4 | 10.170 (8.808, 11.533) | <0.001 | 2.945 (2.183, 3.973) | <0.001 |
| *P*-trend |  | <0.001 |  | <0.001 |
| **VAT volume** |  | | | |
| Continue | 0.013 (0.011, 0.015) | <0.001 | 1.001 (1.001, 1.002) | <0.001 |
| Quantile |  | | | |
| Q1 | 0.00 (Reference) |  | 1.00 (Reference) |  |
| Q2 | 3.269 (2.317, 4.220) | <0.001 | 1.339 (1.104, 1.623) | 0.004 |
| Q3 | 6.563 (5.688, 7.437) | <0.001 | 1.995 (1.615, 2.463) | <0.001 |
| Q4 | 10.170 (8.808, 11.533) | <0.001 | 2.945 (2.183, 3.973) | <0.001 |
| *P*-trend |  | <0.001 |  | <0.001 |
| **VAT area** |  | | | |
| Continue | 0.068 (0.059, 0.078) | <0.001 | 1.007 (1.005, 1.009) | <0.001 |
| Quantile |  |  |  |  |
| Q1 | 0.00 (Reference) |  | 1.00 (Reference) |  |
| Q2 | 3.270 (2.320, 4.221) | <0.001 | 1.340 (1.105, 1.625) | 0.004 |
| Q3 | 6.559 (5.683, 7.436) | <0.001 | 1.992 (1.613, 2.461) | <0.001 |
| Q4 | 10.170 (8.808, 11.532) | <0.001 | 2.944 (2.182, 3.972) | <0.001 |
| *P*-trend |  | <0.001 |  | <0.001 |

Adjusted for age, sex, race, education, status of mariage and poverty, smoking, alcohol consumption, M/VPA, HTN, CVD, cancer, and CKD. VAT, visceral adipose tissue; KDMAge, this is the biological age that is determined according to the Klemera-Doubal method; KDMAgeAccel, KDMAge acceleration; OR, odds ratio; Cl, confidence interval.

**Fig. 1. Restricted cubic splines analyses.** Associations of VAT mass **(A, D)**, VAT volume **(B, E)**, and VAT area **(C, F)** with KDMAge/KDMAgeAccel risk. The calculation of Beta and OR in the figures is performed subsequent to adjustment for age, sex, race, education, marital status, poverty status, smoking status, alcohol consumption, M/VPA, HTN, CVD, cancer, and CKD. VAT, visceral adipose tissue; KDMAge, this is the biological age that is determined according to the Klemera-Doubal method; KDMAgeAccel, KDMAge acceleration; OR, odds ratio; Cl, confidence interval.

References:

1. Tripathi H, Singh A, Farheen, Prakash B, Dubey DK, Sethi P, Jadon RS, Ranjan P, Vikram NK. The Metabolic Score for Visceral Fat (METS-VF) as a predictor of diabetes mellitus: Evidence from the 2011-2018 NHANES study. PLoS One. 2025 Feb 11;20(2):e0317913. doi: 10.1371/journal.pone.0317913. PMID: 39932909; PMCID: PMC11813123.
2. Liao Y, Zhou K, Lin B, Deng S, Weng B, Pan L. Associations between systemic immune-inflammatory index and visceral adipose tissue area: results of a national survey. Front Nutr. 2025 Jan 16;11:1517186. doi: 10.3389/fnut.2024.1517186. PMID: 39885869; PMCID: PMC11780491.
3. Cheng M, Meng Y, Song Z, Zhang L, Zeng Y, Zhang D, Li S. The Association Between Metabolic Score for Visceral Fat and Cognitive Function Among Older Adults in the United States. Nutrients. 2025 Jan 10;17(2):236. doi: 10.3390/nu17020236. PMID: 39861366; PMCID: PMC11768000.
4. Xue M, Zhang X, Chen K, Zheng F, Wang B, Lin Q, Zhang Z, Dong X, Niu W. Visceral adiposity index, premature mortality, and life expectancy in US adults. Lipids Health Dis. 2025 Apr 15;24(1):139. doi: 10.1186/s12944-025-02560-3. PMID: 40234930; PMCID: PMC12001622.
5. Jiang K, Luan H, Pu X, Wang M, Yin J, Gong R. Association Between Visceral Adiposity Index and Insulin Resistance: A Cross-Sectional Study Based on US Adults. Front Endocrinol (Lausanne). 2022 Jul 22;13:921067. doi: 10.3389/fendo.2022.921067. PMID: 35937809; PMCID: PMC9353944.
6. Geng J, Zhang X, Guo Y, Wen H, Guo D, Liang Q, Pu S, Wang Y, Liu M, Li Z, Hu W, Yang X, Chang P, Hu L, Li Y. Moderate-intensity interval exercise exacerbates cardiac lipotoxicity in high-fat, high-calories diet-fed mice. Nat Commun. 2025 Jan 12;16(1):613. doi: 10.1038/s41467-025-55917-8. PMID: 39800728; PMCID: PMC11725574.
7. Xia Z, Shi S, Ma X, Li F, Li X, Gaisano HY, Zhao M, Li Y, He Y, Jiang J. Mediating effect of adiponectin between free fatty acid and tumor necrosis factor-α in patients with diabetes. Nutr Diabetes. 2024 Jun 17;14(1):45. doi: 10.1038/s41387-024-00302-5. PMID: 38886355; PMCID: PMC11183252.

**Responses to Comments of Reviewer #2:**

*Comments 1: The introduction was well-written, but too long. I suggest summarizing it.*

Response: The reviewer's positive appraisal of the Introduction, characterizing it as "well-written," is deeply appreciated. In response to the suggestion to improve conciseness, we have rigorously condensed this section from 734 to 427 words (a 42% reduction). The revised version retains all critical scientific elements while enabling readers to efficiently grasp: (i) the research background, (ii) knowledge gaps, (iii) methodological framework, and (iv) clinical implications. The modified Introduction is presented below:

“The global population is rapidly aging [1]. Epidemiological projections indicate that individuals aged ≥ 60 years will constitute 16% of the global population by 2030, rising to 22% (2.1 billion) by mid-century [2]. Aging is robustly linked to increased risks of multiple chronic conditions, including diabetes mellitus (DM), cardiovascular disease (CVD), hypertension (HTN), chronic kidney disease (CKD), and cancer [3–5]. Given the profound impact of these conditions on healthcare burdens and age-related mortality, accurately assessing biological aging (BA) is crucial for developing interventions to decelerate it. Biological age, a core indicator of physiological decline, provides superior predictive value over chronological age [6]. The Klemera-Doubal method (KDMAge) quantifies BA by integrating multisystem biomarkers (e.g., metabolic, inflammatory, cardiovascular) [7]. Unlike single biomarkers, KDMAge captures individual heterogeneity in aging trajectories through multi-system interactions, serving as an effective predictor of disease risk, functional decline, and mortality [8].

The global overweight/obesity epidemic poses a major public health threat, with prevalence rising exponentially [9]. Obesity independently predicts CVD, HTN, DM, and cancer, ranking as the second leading modifiable mortality risk factor after tobacco in Western populations [10]. In the United States, 70% of adults and 19% of adolescents are affected [11]. Significant sex-based differences exist: females show higher obesity prevalence (18% vs. 14% in males in 2020) with projections indicating persistent disparities (27% vs. 23% by 2035). Adipose distribution also differs markedly, with gluteofemoral predominance in females versus abdominal in males, influenced by biological (e.g., sex hormones) and sociocultural factors [12]. Given these health implications, effective obesity assessment is critical. While body mass index (BMI) and waist circumference (WC) provide unidimensional measures, the visceral adiposity index (VAI) offers a sex-specific metric integrating morphological (BMI, WC) and biochemical markers (triglycerides, HDL). This enables simultaneous evaluation of visceral fat accumulation and metabolic dysfunction, establishing VAI as a validated clinical marker for visceral obesity [13].

Obesity accelerates aging through pathways including vascular dysfunction, insulin resistance (IR), chronic inflammation, and lifestyle factors [14,15]. Of these, IR—clinically measured by homeostatic model assessment (HOMA-IR)—is particularly critical. Obesity impairs insulin signaling via chronic inflammation, lipotoxicity, and metabolic dysregulation [16], while the synergy of obesity and IR promotes age-related comorbidities (e.g., DM, CVD) that exacerbate systemic frailty [17]. However, sex-specific mechanisms mediating the obesity–aging association remain poorly understood, particularly regarding visceral adiposity and IR pathways. Leveraging the National Health and Nutrition Examination Survey (NHANES), the purpose of our investigation was to systematically examine sex-specific associations between VAI and BA while quantifying the mediating role of IR, thereby offering novel insights to inform public health strategies aimed at mitigating obesity-related aging risks.”

*Comments 2: The resolution of the Figures is low.*

Response: Thanks for the feedback on the figure resolution. We agree that high-resolution figures are essential for clarity and meeting journal standards. To address this point comprehensively, we have meticulously processed all figures using the PLOS-specific Preflight Analysis and Conversion Engine (PACE) digital diagnostic tool (<https://pacev2.apexcovantage.com/>). This tool is designed to ensure figures meet *PLOS*'s stringent technical requirements. Following this optimization process, all figures now achieve a resolution of 600 dpi. We are confident that this enhancement significantly improves the clarity and readability of both textual elements and graphical components within the figures. The revised high-resolution figures have been uploaded to the designated “Figures” section in the manuscript submission system. Reviewers can download the original files to verify the improvements. We believe these modifications, prompted by the reviewer's insightful comments, are crucial for fully complying with *PLOS* guidelines and for ensuring that readers can interpret the figures without any difficulty. We are grateful for this constructive suggestion, which has undoubtedly strengthened the presentation quality of our manuscript.

*Comments 3: It would be better to define clinical features, including HTN, DM, CKD, CVD and Cancer.*

Response: We are grateful to the reviewer for their insightful suggestion to elucidate the definitions of clinical covariates. In direct response, the Materials and Methods–Assessment of covariates section has been revised to include standardized diagnostic criteria based on the previous studies (lines 192–200 in the Revised Manuscript with Track Changes): HTN is defined as systolic blood pressure > 140 mmHg or diastolic blood pressure > 90 mmHg or physician diagnosis; CVD encompasses congestive heart failure, coronary artery disease, myocardial infarction, angina pectoris, or stroke; DM requires glycated hemoglobin ≥ 6.5%, fasting glucose ≥ 126 mg/dL, 2-h oral glucose tolerance test ≥ 200 mg/dL, antidiabetic use, or physician confirmation; CKD is identified by estimated glomerular filtration rate < 60 mL/min/1.73m² (CKD-EPI equation) or urine albumin-creatinine ratio > 30 mg/g; cancer is based on self-reported physician-diagnosed malignancy. S2 Table comprehensively documents disease definitions with supporting references, enhancing methodological transparency without sacrificing conciseness.

Thank you very much for your attention and time. We look forward to hearing from you.

Yours sincerely,

Haifeng Liu

E-mail: 18447059019@163.com

July 9, 2025

College of Chinese Medicine, Changchun University of Chinese Medicine, Changchun City, Jilin Province, China
