# Supplementary material for: Sex differences in the association between visceral adiposity index and biological aging: A cross-sectional analysis of NHANES 1999–2018 with mediation by insulin resistance
Source: PLoS One. 2025 Sep 29;20(9):e0333472. doi: 10.1371/journal.pone.0333472 (PMC12478895; doi:10.1371/journal.pone.0333472)
Supplement: Attachment — Submitted filename: Response_to_Reviewers_auresp_2.docx [file pone.0333472.s025.docx]

# Response Letter

Dear PLOS ONE Editorial Team,

We sincerely appreciate the opportunity to submit this revised manuscript entitled ”**Sex differences in the association between visceral adiposity index and biological aging: a cross-sectional analysis of NHANES 1999–2018 with mediation by insulin resistance”** (PONE-D-25-26841R1) to **PLOS ONE**. We extend our deepest gratitude to the editorial team and reviewers for their substantial time investment and insightful critiques, which have significantly strengthened our work.

All reviewer comments have been meticulously addressed through comprehensive revisions. Below, we present the reviewers' comments in italics, followed by our point-by-point responses in **blue** text (with key modifications **highlighted in red**). Supporting documentation appears in standard font, while manuscript changes are tracked using **yellow highlighting**.

The detailed responses follow this section:

**Responses to Comments of Reviewer #3:**

***Comments 1: Given the diverse racial/ethnic composition of the NHANES sample (e.g., Mexican American, Non-Hispanic White, Non-Hispanic Black), it would be valuable to perform subgroup or sensitivity analyses to examine whether the observed associations between VAI, biological aging, and insulin resistance differ across racial/ethnic groups. This could enhance the generalizability and interpretation of your findings.***

Response:

We would like to express our profound gratitude to the reviewer for underscoring the significance of racial/ethnic heterogeneity in our analyses. In response, we conducted comprehensive subgroup analyses across racial/ethnic categories (Mexican American, Non-Hispanic White, Non-Hispanic Black, Other) within the whole cohort and sex-stratified subgroups. Furthermore, three key relationships were examined: (a) associations of visceral adiposity index (VAI) with homeostasis model assessment of insulin resistance (HOMA-IR); (b) relationships between HOMA-IR and biological aging (BA); and (c) associations between VAI and BA.

Key findings:

- All racial/ethnic subgroups consistently demonstrated positive associations mirroring our primary results;
- Significant race/ethnicity interaction effects (*P*_interaction_ < 0.05) emerged for both VAI–HOMA-IR relationships and VAI–BA associations, indicating differential magnitudes of these positive associations across racial/ethnic subgroups.

These findings both confirm the robustness of our primary observations and reveal important population-specific variations. Notably, identifying these differential effects enhances the generalizability of our conclusions and provides valuable insights for targeted interventions. We are grateful for this insightful suggestion, which significantly strengthened our study's epidemiological relevance.

The detailed methodological description (Lines 193–201) and complete analytical results (Lines 422-435) appear in the “Revised Manuscript with Track Changes,” while relevant supporting documentation (Tables S16–S18) can be accessed in the 'Supplementary Information' file.

Revised Manuscript with Track Changes (Lines 193–201):

“To address potential effect modification by demographic factors, we conducted comprehensive subgroup and interaction analyses. Given the racial/ethnic diversity of NHANES participants (Mexican American, Non-Hispanic White, Non-Hispanic Black, Other), we (a) performed race/ethnicity-stratified analyses of VAI–IR, IR–BA and VAI–BA associations; (b) and tested multiplicative interaction terms (VAI × race/ethnicity) in fully adjusted models. Additionally, since age may modify relationships between VAI and BA, we (a) assessed age interaction effects through VAI × age terms; (b) and conducted age-stratified analyses using predefined categories (20–39, 40–59, ≥ 60 years). These analyses were implemented in both the whole cohort and sex-specific subgroups using multivariable-adjusted models. This approach enhances generalizability while identifying population-specific patterns in the visceral adiposity-aging relationship.”

Revised Manuscript with Track Changes (Lines 422–435):

*“*Consistent with our primary findings, stratified analyses across racial/ethnic subgroups (Mexican American, Non-Hispanic White, Non-Hispanic Black, Other) revealed persistently positive associations between VAI, BA, and IR in all populations. Three key patterns emerged: (a) VAI–HOMA-IR associations remained significantly positive across all racial/ethnic groups in the whole cohort and sex-stratified subgroups (S16 Table). Formal interaction testing indicated significant effect modification by race/ethnicity in the whole population (*P*_interaction_ = 0.044) and among females (*P*_interaction_ = 0.018). (b) HOMA-IR–BA relationships demonstrated consistent positive effects in all racial/ethnic subgroups without significant interaction (*P*_interaction_ > 0.05 for whole, male, and female cohorts; S17 Table), indicating race-invariant mediation pathways. (c) The VAI–BA associations maintained significant positive effects across racial/ethnic strata (S18 Table), with significant interaction effects observed in the whole population (KDMAge: *P*_interaction_ = 0.021; KDMAgeAccel: *P*_interaction_ = 0.006) and among males (KDMAge: *P*_interaction_ = 0.025; KDMAgeAccel: *P*_interaction_ = 0.026). These findings confirm the reliability of our primary observations while identifying potential racial/ethnic differences, which enhances the generalizability of the study results and provides a basis for targeted interventions.*”*

Relevant supporting documentation:

**S16 Table. Subgroup analyses of VAI–HOMA-IR associations.**

| Race/Ethnicity | N (%) | **VAI–HOMA-IR associations** | | ***P*** for interaction |
| --- | --- | --- | --- | --- |
|  |  | **β (95% CI)** | ***P***-value |  |
| Whole population |  | | | |
| Mexican American | 3357 (17.23) | 0.47 (0.31–0.63) | <0.001 | 0.044 |
| Non-Hispanic White | 8996 (46.17) | 0.40 (0.30–0.50) | <0.001 |  |
| Non-Hispanic Black | 3803 (19.52) | 1.15 (0.77–1.52) | <0.001 |  |
| Other | 3330 (17.09) | 0.33 (0.05–0.61) | 0.023 |  |
| Females |  | | | |
| Mexican American | 1641 (16.86) | 0.54 (0.30–0.78) | <0.001 | 0.018 |
| Non-Hispanic White | 4420 (45.42) | 0.45 (0.27–0.64) | <0.001 |  |
| Non-Hispanic Black | 1966 (20.20) | 1.33 (0.76–1.90) | <0.001 |  |
| Other | 1705 (17.52) | 0.53 (0.24–0.81) | <0.001 |  |
| Males |  | | | |
| Mexican American | 1716 (17.59) | 0.41 (0.18–0.64) | <0.001 | 0.204 |
| Non-Hispanic White | 4576 (46.91) | 0.36 (0.25–0.47) | <0.001 |  |
| Non-Hispanic Black | 1837 (18.83) | 1.01 (0.55–1.47) | <0.001 |  |
| Other | 1625 (16.66) | 0.23 (-0.06–0.52) | 0.118 |  |

The models were adjusted for age, sex (only in the model of the whole population), education, marital status, poverty status, smoking status, alcohol consumption, M/VPA, HTN, CVD, cancer, and CKD. HOMA-IR, homeostasis model assessment of insulin resistance; VAI, visceral adiposity index; CI, confidence interval.

**S17 Table. Subgroup analyses of HOMA-IR–BA associations.**

| Race/Ethnicity | N (%) | **HOMA-IR–KDMAge associations** | | ***P* for interaction** | **HOMA-IR–KDMAgeAccel associations** | | ***P* for interaction** |
| --- | --- | --- | --- | --- | --- | --- | --- |
|  |  | **β (95% CI)** | ***P*-value** |  | **OR (95% CI)** | ***P*-value** |  |
| Whole population |  | | | | | | |
| Mexican American | 3357 (17.23) | 0.20 (0.01–0.42) | 0.007 | 0.481 | 1.05 (1.01–1.11) | 0.011 | 0.609 |
| Non-Hispanic White | 8996 (46.17) | 0.37 (0.28–0.47) | <0.001 |  | 1.08 (1.06–1.11) | <0.001 |  |
| Non-Hispanic Black | 3803 (19.52) | 0.34 (0.22–0.46) | <0.001 |  | 1.05 (1.02–1.08) | 0.001 |  |
| Other | 3330 (17.09) | 0.38 (0.17–0.58) | <0.001 |  | 1.07 (1.03–1.12) | 0.001 |  |
| Females |  | | | | | | |
| Mexican American | 1641 (16.86) | 0.19 (-0.03–0.41) | 0.091 | 0.479 | 1.06 (1.01–1.11) | 0.016 | 0.816 |
| Non-Hispanic White | 4420 (45.42) | 0.37 (0.23–0.51) | <0.001 |  | 1.08 (1.04–1.12) | <0.001 |  |
| Non-Hispanic Black | 1966 (20.20) | 0.30 (0.16–0.44) | <0.001 |  | 1.05 (1.01–1.09) | 0.011 |  |
| Other | 1705 (17.52) | 0.42 (0.21–0.62) | <0.001 |  | 1.07 (1.02–1.13) | 0.013 |  |
| Males |  | | | | | | |
| Mexican American | 1716 (17.59) | 0.19 (-0.11–0.49) | 0.219 | 0.560 | 1.04 (0.95–1.13) | 0.407 | 0.732 |
| Non-Hispanic White | 4576 (46.91) | 0.37 (0.24–0.50) | <0.001 |  | 1.08 (1.05–1.11) | <0.001 |  |
| Non-Hispanic Black | 1837 (18.83) | 0.38 (0.17–0.59) | <0.001 |  | 1.05 (1.00–1.10) | 0.06 |  |
| Other | 1625 (16.66) | 0.35 (0.13–0.57) | 0.002 |  | 1.08 (1.02–1.14) | 0.011 |  |

The models were adjusted for age, sex (only in the model of the whole population), education, marital status, poverty status, smoking status, alcohol consumption, M/VPA, HTN, CVD, cancer, and CKD. HOMA-IR, homeostasis model assessment of insulin resistance; KDMAge, Klemera-Doubal method age; KDMAgeAccel, KDMAge acceleration; CI, confidence interval.

**S18 Table. Subgroup analyses of VAI–BA associations for different race/ethnicity.**

| **Race/Ethnicity** | **N (%)** | **VAI–KDMAge associations** | | ***P* for interaction** | **VAI–KDMAgeAccel associations** | | ***P* for interaction** |
| --- | --- | --- | --- | --- | --- | --- | --- |
|  |  | **β (95% CI)** | ***P*-value** |  | **OR (95% CI)** | ***P*-value** |  |
| Whole population |  | | | | | | |
| Mexican American | 3357 (17.23) | 0.64 (0.40–0.88) | <0.001 | 0.021 | 1.08 (1.03–1.13) | 0.002 | 0.006 |
| Non-Hispanic White | 8996 (46.17) | 0.85 (0.68–1.01) | <0.001 |  | 1.16 (1.13–1.20) | <0.001 |  |
| Non-Hispanic Black | 3803 (19.52) | 1.15 (0.64–1.66) | <0.001 |  | 1.15 (1.06–1.25) | 0.001 |  |
| Other | 3330 (17.09) | 0.43 (0.15–0.71) | 0.003 |  | 1.08 (1.03–1.12) | <0.001 |  |
| Females |  | | | | | | |
| Mexican American | 1641 (16.86) | 0.77 (0.42–1.13) | <0.001 | 0.334 | 1.18 (1.08–1.28) | <0.001 | 0.642 |
| Non-Hispanic White | 4420 (45.42) | 1.04 (0.78–1.30) | <0.001 |  | 1.24 (1.18–1.31) | <0.001 |  |
| Non-Hispanic Black | 1966 (20.20) | 1.48 (0.88–2.08) | <0.001 |  | 1.22 (1.10–1.36) | <0.001 |  |
| Other | 1705 (17.52) | 0.92 (0.42–1.41) | <0.001 |  | 1.14 (1.06–1.23) | <0.001 |  |
| Males |  | | | | | | |
| Mexican American | 1716 (17.59) | 0.53 (0.22–0.84) | 0.001 | 0.025 | 1.04 (0.99–1.09) | 0.141 | 0.026 |
| Non-Hispanic White | 4576 (46.91) | 0.73 (0.50–0.95) | <0.001 |  | 1.13 (1.08–1.18) | <0.001 |  |
| Non-Hispanic Black | 1837 (18.83) | 0.84 (0.13–1.56) | 0.023 |  | 1.09 (0.99–1.20) | 0.095 |  |
| Other | 1625 (16.66) | 0.27 (0.01–0.52) | 0.041 |  | 1.06 (1.02–1.11) | 0.005 |  |

The models were adjusted for age, sex (only in the model of the whole population), education, marital status, poverty status, smoking status, alcohol consumption, M/VPA, HTN, CVD, cancer, and CKD. VAI, visceral adiposity index; KDMAge, Klemera-Doubal method age; KDMAgeAccel, KDMAge acceleration; CI, confidence interval.

***Comments 2: While the cross-sectional design limitation is acknowledged, I recommend elaborating on its implications, particularly regarding causal inference and temporality. Discussing how reverse causation might influence the observed relationships would improve the depth of the discussion.***

Response:

We are grateful to the reviewer for the thoughtful feedback regarding the limitations of our cross-sectional design. In direct response to this comment, we have substantially expanded the limitations of the Discussion section (originally 38 words → now 432 words), incorporating six additional references. Specifically, we have (a) analysed the possibility of reverse causation in the observed associations, and delineated potential biological mechanisms through which accelerated BA might drive visceral adiposity accumulation; (b) critically examined how such reverse causation could influence the interpretation of our observed associations. This revision significantly enhances the depth of our methodological discussion and provides important context for interpreting the findings. We believe these additions will allow readers to more objectively evaluate our results while strengthening the scientific rigor of our conclusions.

The revised text appears in Lines 541-576 of the “Revised Manuscript with Track Changes”.

Revised Manuscript with Track Changes (Lines 541–576):

“First, the primary limitation of this study stems from its cross-sectional design, which inherently precludes definitive causal inference regarding the temporal sequence between VAI and BA, particularly in elucidating sex-specific mechanisms. While our analyses revealed significant associations and mediation effects, this design cannot establish whether elevated VAI precedes and contributes to accelerated BA, or conversely, whether accelerated BA itself drives increases in VAI. Compelling evidence suggests that age-related lipid turnover decline and adipose tissue dysfunction may constitute reverse causal pathways. Specifically, longitudinal data demonstrate that adipose lipid turnover decreases by approximately 4.7% annually with aging, disrupting the dynamic equilibrium of lipid storage and clearance, even under constant caloric intake, potentially driving weight gain exceeding 20% over decades [55]. This dysregulation is particularly pronounced in visceral adipose tissue due to diminished preadipocyte differentiation capacity, reduced lipolytic enzyme activity (e.g., hormone-sensitive lipase), and blunted catecholamine responsiveness, collectively impairing lipid mobilization. Notably, the recent identification of CP-A adipose progenitor cells, a subpopulation emerging during middle age that exhibits hyperactive adipogenic potential through LIFR-STAT3 signaling activation, may further explain age-dependent visceral adipose tissue expansion [56]. Concurrently, adipose tissue senescence is characterized by chronic low-grade inflammation involving increased immune cell infiltration (e.g., TNF-α, IL-6), tissue fibrosis, and adipokine dysregulation (e.g., reduced adiponectin, elevated leptin), establishing a self-perpetuating vicious cycle whereby aging promotes adipose dysfunction that subsequently exacerbates systemic metabolic deterioration and accelerates organismal decline [57]. Additional contributors to age-associated visceral adiposity include muscle mass loss, progressive reductions in physical activity levels, and hormonal alterations [11,58,59]. Critically, the observed mediation proportion of HOMA-IR, while statistically significant, must also be interpreted cautiously within this bidirectional framework, as aging-related metabolic dysfunction may simultaneously elevate HOMA-IR independently of prior adiposity changes [60]. Importantly, this potential reverse causation may influence our key finding of stronger VAI–BA associations in females. Estrogen, predominantly in premenopausal women, maintains subcutaneous fat distribution and functionality while delaying visceral adipose tissue accumulation through ERα-mediated mechanisms; however, the abrupt estrogen decline during menopause may precipitate rapid visceral adipogenesis, potentially amplifying the observed VAI–BA relationship in female cohorts [59]. Furthermore, the biphasic nonlinear associations and threshold effects we identified may reflect shifting dominance between causal directions: below VAI thresholds, traditional “adiposity-driven aging” mechanisms (lipotoxicity, inflammation) likely predominate, whereas above thresholds, aging-induced metabolic dysregulation may progressively disrupt adipose homeostasis, creating a self-reinforcing pathological loop [57]. Consequently, the strength and sex-specific patterns of the associations reported here require validation in longitudinal studies tracking temporal dynamics of VAI, HOMA-IR, and BA biomarkers to establish precedence. Future experimental interventions targeting visceral adiposity reduction would provide stronger evidence for causal pathways and their sexual dimorphism.”

References:

11.	Nunan E, Wright CL, Semola OA, Subramanian M, Balasubramanian P, Lovern PC, et al. Obesity as a premature aging phenotype — implications for sarcopenic obesity. GeroScience. 2022;44: 1393–1405. doi:10.1007/s11357-022-00567-7

55.	Arner P, Bernard S, Appelsved L, Fu K-Y, Andersson DP, Salehpour M, et al. Adipose lipid turnover and long-term changes in body weight. Nat Med. 2019;25: 1385–1389. doi:10.1038/s41591-019-0565-5

56.	Wang G, Li G, Song A, Zhao Y, Yu J, Wang Y, et al. Distinct adipose progenitor cells emerging with age drive active adipogenesis. Science. 2025;388: eadj0430. doi:10.1126/science.adj0430

57.	Nguyen TT, Corvera S. Adipose tissue as a linchpin of organismal ageing. Nat Metab. 2024;6: 793–807. doi:10.1038/s42255-024-01046-3

58.	Winters-van Eekelen E, van der Velde JHPM, Boone SC, Westgate K, Brage S, Lamb HJ, et al. Objectively Measured Physical Activity and Body Fatness: Associations with Total Body Fat, Visceral Fat, and Liver Fat. Med Sci Sports Exerc. 2021;53: 2309–2317. doi:10.1249/MSS.0000000000002712

59.	Vecchiatto B, Castro TL, Ferreira NJR, Evangelista FS. Healthy adipose tissue after menopause: contribution of balanced diet and physical exercise. Explor Endocr Metab Dis. 2025; 101424. doi:10.37349/eemd.2025.101424

60.	Palmer AK, Jensen MD. Metabolic changes in aging humans: current evidence and therapeutic strategies. J Clin Invest. 132: e158451. doi:10.1172/JCI158451

***Comments 3: Age may act as a potential effect modifier in the relationship between VAI and biological aging. Please consider testing for an interaction between age and VAI, and if significant, stratifying the analyses accordingly. This may provide important age-related insights into the adiposity-aging relationship.***

Response:

We appreciate the reviewer for the valuable insights concerning the potential effects related to age. To investigate the possible effect of age on VAI–BA relationship, the following operations were carried out in the results section: (a) Implemented multiplicative interaction testing (VAI × age group) in the whole cohort and sex-stratified subgroups, using predefined age categories (20–39, 40–59, ≥ 60 years); (b) Upon detecting significant age interaction in the whole cohort (KDMAge: *P*_interaction_ = 0.017; KDMAgeAccel: *P*_interaction_ = 0.039), we conducted stratified analyses; (c) Performed age-stratified analyses in all cohorts.

Key findings:

- All age subgroups maintained significant positive VAI–BA associations (*P* < 0.05), confirming robustness across a wide age range.
- Young adults (20–39 years) exhibited the strongest effects: 1.05-year KDMAge acceleration per VAI unit (β = 1.05, 95% CI: 0.83-1.28; *P* < 0.001); 22% higher KDMAgeAccel risk per VAI unit (OR = 1.22, 95% CI: 1.16-1.29; *P* < 0.001)
- No significant age interactions were observed in sex-specific analyses (*P*_interaction_ > 0.05)

These results reveal a previously unrecognized vulnerability of younger adults to visceral adiposity-induced BA, highlighting critical age windows for preventive intervention. We are grateful for this valuable suggestion, which uncovered important life-course patterns in adiposity–aging relationships.

Full results appear in the “Revised Manuscript with Track Changes” (Lines 436–445) and S19Table .

Revised Manuscript with Track Changes (Lines 436–445):

“A subsequent age-stratified analysis of all subgroups (20–39 years, 40–59 years, ≥60 years) demonstrated a significant positive association between VAI and BA in all three cohorts (*P* < 0.05 for all age groups; S19 Table). This finding is aligned with the conclusions drawn from our preliminary analysis. Formal interaction testing revealed significant effect modification by age in the whole population for both KDMAge (*P*_interaction_ = 0.017) and KDMAgeAccel risk (*P*_interaction_ = 0.039). Notably, the strongest associations emerged in young adults (20-39 years), where each unit VAI increase corresponded to 1.05-year KDMAge (β = 1.05, 95% CI: 0.83-1.28; *P* < 0.001) and 22% higher KDMAgeAccel risk (OR = 1.22, 95% CI: 1.16-1.29; *P* < 0.001). Surprisingly, sex-stratified analyses showed no significant age interaction in either male or female subgroups (*P*_interaction_ > 0.05). These findings reveal a previously unrecognized vulnerability of younger adults to visceral adiposity-induced BA, highlighting critical windows for preventive intervention.”

Relevant supporting documentation:

**S19 Table. Subgroup analyses of VAI–BA associations for different age groups.**

| **Age groups (years)** | **N (%)** | **VAI–KDMAge associations** | | ***P* for interaction** | **VAI–KDMAgeAccel associations** | | ***P* for interaction** |
| --- | --- | --- | --- | --- | --- | --- | --- |
|  |  | **β (95% CI)** | ***P*-value** |  | **OR (95% CI)** | ***P*-value** |  |
| Whole population |  | | | | | | |
| 20–39 | 6358 (32.63) | 1.05 (0.83–1.28) | <0.001 | 0.017 | 1.22 (1.16–1.29) | <0.001 | 0.039 |
| 40–59 | 6527 (33.50) | 0.64 (0.49–0.79) | <0.001 |  | 1.11 (1.07–1.16) | <0.001 |  |
| ≥ 60 | 6601 (33.88) | 0.85 (0.56–1.14) | <0.001 |  | 1.15 (1.09–1.21) | <0.001 |  |
| Females |  | | | | | | |
| 20–39 | 3148 (32.35) | 1.29 (0.91–1.67) | <0.001 | 0.548 | 1.40 (1.27–1.54) | <0.001 | 0.209 |
| 40–59 | 3298 (33.89) | 0.93 (0.60–1.25) | <0.001 |  | 1.18 (1.10–1.27) | <0.001 |  |
| ≥ 60 | 3286 (33.76) | 1.01 (0.69–1.33) | <0.001 |  | 1.19 (1.12–1.26) | <0.001 |  |
| Males |  | | | | | | |
| 20–39 | 3210 (32.91) | 0.91 (0.60–1.21) | <0.001 | 0.052 | 1.15 (1.08–1.22) | <0.001 | 0.335 |
| 40–59 | 3229 (33.10) | 0.46 (0.31–0.61) | <0.001 |  | 1.08 (1.04–1.13) | <0.001 |  |
| ≥ 60 | 3315 (33.99) | 0.71 (0.18–1.25) | 0.010 |  | 1.10 (1.02–1.19) | 0.013 |  |

The models were adjusted for sex (only in the model of the whole population), race, education, marital status, poverty status, smoking status, alcohol consumption, M/VPA, HTN, CVD, cancer, and CKD. VAI, visceral adiposity index; KDMAge, Klemera-Doubal method age; KDMAgeAccel, KDMAge acceleration; CI, confidence interval.

***Comments 4: Given the strong association between diabetes and both insulin resistance and biological aging, a sensitivity analysis excluding individuals with diagnosed diabetes could help evaluate whether the observed associations are independent of overt diabetic status.***

Response:

We are grateful for the reviewer's perspicacious observation regarding the possible confounding effect of overt diabetes. To evaluate whether the observed associations are independent of diabetic status, we performed additional sensitivity analyses excluding all individuals with diagnosed diabetes mellitus (n = 3708, 19.03%). Using the same analytical framework applied in our primary analysis, we re-examined the associations between VAI and BA, assessed via Klemera-Doubal method age (KDMAge) and KDMAge acceleration (KDMAgeAccel) risk, in the whole population and sex-stratified subgroups. This included multivariable regression, restricted cubic splines (RCSs), and mediation analyses. The additional sensitivity analyses rigorously confirmed the robustness of our primary findings:

- The positive VAI–BA associations remained statistically significant;
- Sex-specific patterns persisted (stronger associations in females, greater HOMA-IR mediation in males).

These outcomes demonstrate that the reported associations operate independently of overt diabetic status. We thank the reviewer for this valuable suggestion, which strengthened our study's robustness.

Full results appear in the “Revised Manuscript with Track Changes” (Lines 397–419) and relevant supporting documentation (S12–S15 Tables and S2 Fig).

Revised Manuscript with Track Changes (Lines 397–419):

“To evaluate the potential confounding effect of overt diabetes, sensitivity analyses were performed after excluding all individuals with diagnosed DM (n=3708, 19.03%). VAI maintained robust positive associations with BA in the whole population (β_KDMAge_ = 0.77, 95% CI: 0.62–0.93; OR_KDMAgeAccel_ = 1.15, 95% CI: 1.12–1.18; P < 0.001), with significant sex interaction effects (*P*_interaction_ < 0.01). Sex-stratified analyses revealed significantly stronger associations in females than males for both continuous VAI (KDMAge: β_female_ = 1.13, 95% CI: 0.81–1.46 vs. β_male_ = 0.59, 95% CI: 0.43–0.75; KDMAgeAccel: OR_female_ = 1.27, 95% CI: 1.21–1.34 vs. OR_male_ = 1.10, 95% CI: 1.06–1.14; P < 0.001) and highest VAI quartile comparisons (KDMAge: β_female_ = 6.97, 95% CI: 5.97–7.96 vs. β_male_ = 5.97, 95% CI: 4.77–7.17; KDMAgeAccel: OR_female_ = 3.46, 95% CI: 2.80–4.27 vs. OR_male_ = 2.43, 95% CI: 1.96–3.02; P < 0.001). Significant linear trends persisted across VAI quartiles in both sexes (*P*_trend_ < 0.001). Complete results are presented in S12 and S13 Tables.

Re-evaluated RCSs (S2 Fig) and threshold analyses (S14 Table) confirmed the persistence of sex-specific patterns in VAI–BA relationships. Females demonstrated stronger associations with KDMAge across a broader VAI range (inflection point: K_female_ = 3.41 vs. K_male_ = 2.51), despite comparable effect magnitudes both below (β_female_ = 2.48, 95% CI: 2.12–2.84 vs. β_male_ = 2.35, 95% CI: 1.73–2.96; P < 0.001) and above thresholds (β_female_ = 0.18, 95% CI: 0.02–0.39 vs. β_male_ = 0.24, 95% CI: 0.08–0.39; P < 0.01). Similarly, for KDMAgeAccel risk, females exhibited markedly lower threshold inflection points (K_female_  = 0.60 vs. K_male_  = 2.53) with stronger associations above the threshold (OR_female_ = 1.18, 95% CI: 1.14–1.22 vs. OR_male_ = 1.04, 95% CI: 1.02–1.07; P < 0.01).

Mediation analyses (S15 Table) revealed consistent sex differences in HOMA-IR mediation proportions after excluding diabetic participants. HOMA-IR mediated 17.85% of the VAI–KDMAge association and 22.59% of the VAI–KDMAgeAccel risk association in the whole population. Mediation proportions remained significantly higher in males than females for both KDMAge (21.56% vs. 11.11%) and KDMAgeAccel risk (29.46% vs. 18.98%). All indirect effects remained statistically significant (P < 0.001).”

Relevant supporting documentation:

**S12 Table. Sex interaction analysis following exclusion of DM participants.**

|  | **Associations between VAI and KDMAge** | | | **Associations between VAI and KDMAgeAccel risk** | | |
| --- | --- | --- | --- | --- | --- | --- |
|  | **β (95% CI)** | ***P*-value** | ***P* for interaction** | **OR (95% CI)** | ***P*-value** | ***P* for interaction** |
| Females | 1.13 (0.81 ~ 1.46) | <0.001 | 0.007 | 1.27 (1.21 ~ 1.34) | <0.001 | <0.001 |
| Males | 0.59 (0.43 ~ 0.75) | <0.001 |  | 1.10 (1.06 ~ 1.14) | <0.001 |  |

DM, diabetes mellitus; VAI, visceral adiposity index; KDMAge, Klemera-Doubal method age; KDMAgeAccel, KDMAge acceleration; CI, confidence interval; OR, odds ratio. The models were adjusted for age, race, education, marital status, poverty status, smoking status, alcohol consumption, M/VPA, HTN, CVD, cancer, and CKD.

**S13 Table. Multivariate regression analysis following exclusion of DM participants.**

|  | **Associations between VAI and KDMAge** | | **Associations between VAI and KDMAgeAccel risk** | |
| --- | --- | --- | --- | --- |
|  | **β (95% CI)** | ***P***-value | **OR (95% CI)** | ***P***-value |
| Whole pupulation |  | | | |
| VAI continue | 0.77 (0.62–0.93) | <0.001 | 1.15 (1.12–1.18) | <0.001 |
| VAI quantile |  | | | |
| Q1 | 0.00 (Reference) |  | 1.00 (Reference) |  |
| Q2 | 1.47 (0.82–2.11) | <0.001 | 1.34 (1.18–1.53) | <0.001 |
| Q3 | 3.44 (2.69–4.20) | <0.001 | 1.77 (1.50–2.09) | <0.001 |
| Q4 | 6.11 (5.30–6.92) | <0.001 | 2.70 (2.33–3.13) | <0.001 |
| *P*-trend |  | <0.001 |  | <0.001 |
| Females |  | | | |
| VAI continue | 1.13 (0.81–1.46) | <0.001 | 1.27 (1.21–1.34) | <0.001 |
| VAI quantile |  | | | |
| Q1 | 0.00 (Reference) |  | 1.00 (Reference) |  |
| Q2 | 1.53 (0.80–2.27) | <0.001 | 1.36 (1.13–1.65) | 0.002 |
| Q3 | 3.76 (2.90–4.62) | <0.001 | 2.07 (1.67–2.55) | <0.001 |
| Q4 | 6.97 (5.97–7.96) | <0.001 | 3.46 (2.80–4.27) | <0.001 |
| *P*-trend |  | <0.001 |  | <0.001 |
| Males |  | | | |
| VAI continue | 0.59 (0.43–0.75) | <0.001 | 1.10 (1.06–1.14) | <0.001 |
| VAI quantile |  | | | |
| Q1 | 0.00 (Reference) |  | 1.00 (Reference) |  |
| Q2 | 2.08 (1.05–3.10) | <0.001 | 1.46 (1.19–1.79) | <0.001 |
| Q3 | 3.55 (2.41–4.70) | <0.001 | 1.61 (1.27–2.04) | <0.001 |
| Q4 | 5.97 (4.77–7.17) | <0.001 | 2.43 (1.96–3.02) | <0.001 |
| *P*-trend |  | <0.001 |  | <0.001 |

The models were adjusted for age, sex (only in the model of the whole population), race, education, marital status, poverty status, smoking status, alcohol consumption, M/VPA, HTN, CVD, cancer, and CKD. DM, diabetes mellitus; VAI, visceral adiposity index; KDMAge, Klemera-Doubal method age; KDMAgeAccel, KDMAge acceleration; CI, confidence interval; OR, odds ratio.

**S14 Table. Threshold effect analysis following exclusion of DM participants.**

|  | **Associations between VAI and KDMAge** | | **Associations between VAI and KDMAgeAccel risk** | |
| --- | --- | --- | --- | --- |
|  | **β (95% CI)** | ***P*-value** | **OR (95% CI)** | ***P*-value** |
| Whole population |  | | | |
| Standard linear regression | 0.71 (0.62–0.80) | <0.001 | 1.12 (1.10–1.15) | <0.001 |
| Two-piecewise linear regression |  | | | |
| K | 2.845 |  | 2.543 |  |
| <K | 2.34 (2.00–2.68) | <0.001 | 1.50 (1.39–1.62) | <0.001 |
| ≥K | 0.24 (0.12–0.36) | <0.001 | 1.04 (1.02–1.06) | <0.001 |
| Likelihood ratio |  | <0.001 |  | <0.001 |
| Females |  | | | |
| Standard linear regression | 0.96 (0.83–1.09) | <0.001 | 1.20 (1.16–1.24) | <0.001 |
| Two-piecewise linear regression |  | | | |
| K | 3.413 |  | 0.603 |  |
| <K | 2.48 (2.12–2.84) | <0.001 | 1.07 (0.20–5.66) | 0.941 |
| ≥K | 0.18 (0.02–0.39) | 0.004 | 1.18 (1.14–1.22) | <0.001 |
| Likelihood ratio |  | <0.001 |  | <0.001 |
| Males |  | | | |
| Standard linear regression | 0.59 (0.46–0.71) | <0.001 | 1.09 (1.07–1.12) | <0.001 |
| Two-piecewise linear regression |  | | | |
| K | 2.512 |  | 2.528 |  |
| <K | 2.35 (1.73–2.96) | <0.001 | 1.35 (1.22–1.51) | <0.001 |
| ≥K | 0.24 (0.08–0.39) | 0.003 | 1.04 (1.02–1.07) | 0.002 |
| Likelihood ratio |  | <0.001 |  | <0.001 |

The models were adjusted for age, sex (only in the model of the whole population), race, education, marital status, poverty status, smoking status, alcohol consumption, M/VPA, HTN, CVD, cancer, and CKD. DM, diabetes mellitus; VAI, visceral adiposity index; KDMAge, Klemera-Doubal method age; KDMAgeAccel, KDMAge acceleration; CI, confidence interval; OR, odds ratio.

**S15 Table. Mediation analysis following exclusion of DM participants.**

|  | | **Whole Population** | | | **Females** | | | **Males** | | |
| --- | --- | --- | --- | --- | --- | --- | --- | --- | --- | --- |
|  |  | **β (95% CI)** | ***P*-value** | **Mediation Proportion (%)** | **β (95% CI)** | ***P*-value** | **Mediation Proportion (%)** | **β (95% CI)** | ***P*-value** | **Mediation Proportion (%)** |
| DKMAge | Indirect | 0.146 (0.123–0.191) | <0.001 | 17.85 | 0.156 (0.101–0.203) | <0.001 | 11.11 | 0.139 (0.111–0.203) | <0.001 | 21.56 |
|  | Direct | 0.674 (0.501–0.767) | <0.001 |  | 1.247 (0.754–1.250) | <0.001 |  | 0.504 (0.302–0.594) | <0.001 |  |
|  | Total | 0.820 (0.639–0.938) | <0.001 |  | 1.403 (0.876–1.415) | <0.001 |  | 0.643 (0.436–0.768) | <0.001 |  |
| DKMAgeAccel  risk | Indirect | 0.006 (0.005–0.007) | <0.001 | 22.59 | 0.007 (0.004–0.009) | <0.001 | 18.98 | 0.005 (0.004–0.007) | <0.001 | 29.46 |
|  | Direct | 0.020 (0.012–0.021) | <0.001 |  | 0.032 (0.024–0.039) | <0.001 |  | 0.013 (0.006–0.015) | <0.001 |  |
|  | Total | 0.026 (0.018–0.027) | <0.001 |  | 0.039 (0.031–0.045) | <0.001 |  | 0.018 (0.012–0.021) | <0.001 |  |

The models were adjusted for age, sex (only in the model of the whole population), race, education, marital status, poverty status, smoking status, alcohol consumption, M/VPA, HTN, CVD, cancer, and CKD. DM, diabetes mellitus; KDMAge, Klemera-Doubal method age; KDMAgeAccel, KDMAge acceleration; CI, confidence interval.

**S2 Fig. Restricted cubic splines analyses following exclusion of DM participants.** Associations between VAI and KDMAge/KDMAgeAccel risk among the whole population (**A, D**), the females (**B, E**), and the males (**C, F**). The calculation of Beta and OR in the figures is performed subsequent to adjustment for age, sex (only in the model of whole population), race, education, marital status, poverty status, smoking status, alcohol consumption, M/VPA, HTN, CVD, cancer, and CKD. DM, diabetes mellitus; VAI, visceral adiposity index; KDMAge, this is the biological age that is determined according to the Klemera-Doubal method; KDMAgeAccel, KDMAge acceleration; OR, odds ratio; Cl, confidence interval.

***Comments 5: The conclusion could be expanded to highlight the potential clinical and public health implications of your findings. For example, discussing how sex-specific strategies targeting visceral adiposity may mitigate aging-related health risks would underscore the translational value of the study.***

Response:

We appreciate the reviewer's recognition of our work's translational potential. In direct response, we have comprehensively revised the Conclusion section to explicitly highlight both clinical and public health implications. The expanded text now:

- Details sex-specific clinical strategies targeting visceral adiposity, including differentiated intervention approaches for women and men based on our threshold and mediation findings;
- Proposes actionable public health applications such as sex-stratified screening protocols and community-based programs;
- Emphasizes how these strategies could mitigate aging-related health risks.

These additions substantially enhance the practical relevance of our work and directly address the reviewer's valuable point about underscoring translational significance.

The revised conclusion now appears in Lines 589-603 of the “Revised Manuscript with Track Changes”.

Revised Manuscript with Track Changes (Lines 589–603):

“Our findings demonstrate a significant positive association between VAI and BA, with a stronger correlation observed in females and a greater proportion of this association mediated by HOMA-IR in males. These findings have critical translational implications **for developing sex-tailored public health strategies to mitigate obesity-related aging risks. Clinically, our identification of nonlinear thresholds provides potential screening targets for early intervention. In females, who demonstrate** heightened susceptibility to visceral adiposity impacts **even at comparable adiposity levels, preventive measures should focus on midlife adiposity monitoring with intensified lifestyle interventions during critical windows like menopausal transition. In males,** for whom IR mediates **over 20% of the VAI–BA relationship, clinical approaches could prioritize metabolic health optimization through IR-targeted therapies alongside visceral fat reduction. Public health initiatives could incorporate these insights** through**: (a)** development of sex-stratified VAI thresholds **in aging risk algorithms; (b)** tailored community-based programs **(e.g., women-focused visceral fat reduction groups, men-centered metabolic health workshops); and (c)** early implementation of life-course interventions **in high-risk populations identified through these biomarkers. Future longitudinal studies should validate these thresholds and test whether sex-specific reductions in visceral adiposity effectively** slow progression of BA, **potentially informing precision prevention frameworks for aging-related** health risks**.”**

We are truly grateful to the editorial team and reviewers for their thorough evaluation and insightful feedback, which have significantly enhanced our manuscript. The constructive feedback has been incredibly beneficial, enhancing the scientific rigor of our work and expanding its translational relevance. We are extremely grateful for the time, expertise, and thoughtful engagement demonstrated throughout this review process. The revised manuscript now addresses all the concerns raised and demonstrates significantly enhanced scientific rigor. We believe that the current version meets the journal's publication standards and hope that this manuscript will be accepted and published. We are extremely grateful for your consideration of our work and for advancing this vital discussion on sex-specific approaches to mitigating obesity-related aging risks.

Thank you very much for your attention and time. We look forward to hearing from you.

Yours sincerely,

Haifeng Liu

E-mail: 18447059019@163.com

August 10, 2025

College of Chinese Medicine, Changchun University of Chinese Medicine, Changchun City, Jilin Province, China
